# A cortical immune network map identifies distinct microglial transcriptional programs associated with β-amyloid and Tau pathologies

**DOI:** 10.1038/s41398-020-01175-9

**Published:** 2021-01-14

**Authors:** Ellis Patrick, Marta Olah, Mariko Taga, Hans-Ulrich Klein, Jishu Xu, Charles C. White, Daniel Felsky, Sonal Agrawal, Chris Gaiteri, Lori B. Chibnik, Sara Mostafavi, Julie A. Schneider, David A. Bennett, Elizabeth M. Bradshaw, Philip L. De Jager

**Affiliations:** 1 School of Mathematics and Statistics, The University of Sydney, Sydney, NSW Australia; 2 The Westmead Institute for Medical Research, The University of Sydney, Sydney, NSW Australia; 3 Center for Translational & Computational Neuroimmunology, Department of Neurology, Columbia University Medical Center, New York, NY USA; 4 Taub Institute for Research on Alzheimer’s Disease and the Aging Brain, Columbia University Irving Medical Center, New York, NY USA; 5 Rush Alzheimer’s Disease Center, Rush University Medical Center, Chicago, IL USA; 6 Cell Circuits Program, Broad Institute, Cambridge, MA USA; 7 Krembil Centre for Neuroinformatics, Centre for Addiction and Mental Health, Toronto, ON Canada; 8 Department of Psychiatry & Institute of Medical Science, University of Toronto, Toronto, ON Canada; 9 Department of Neurology, Massachusetts General Hospital, Charlestown, MA USA; 10 Department of Epidemiology, Harvard TH Chan School of Public Health, Boston, MA USA; 11 Stanley Center for Psychiatric Genetics, Broad Institute of MIT and Harvard, Cambridge, MA USA; 12 Paul Allen School of Computer Science and Engineering, University of Washington, Seattle, WA USA

**Keywords:** Molecular neuroscience, Comparative genomics

## Abstract

Microglial dysfunction has been proposed as one of the many cellular mechanisms that can contribute to the development of Alzheimer’s disease (AD). Here, using a transcriptional network map of the human frontal cortex, we identify five modules of co-expressed genes related to microglia and assess their role in the neuropathologic features of AD in 540 subjects from two cohort studies of brain aging. Two of these transcriptional programs—modules 113 and 114—relate to the accumulation of β-amyloid, while module 5 relates to tau pathology. We replicate these associations in brain epigenomic data and in two independent datasets. In terms of tau, we propose that module 5, a marker of activated microglia, may lead to tau accumulation and subsequent cognitive decline. We validate our model further by showing that three representative module 5 genes (*ACADVL, TRABD*, and *VASP*) encode proteins that are upregulated in activated microglia in AD.

## Introduction

Alzheimer’s disease (AD) is characterized pathologically by the accumulation of both β-amyloid and Tau pathologies which lead to the gradual loss of cognitive function and, ultimately, dementia^1^. The amount of these two pathologies that are present in the older brain is strongly but only partially correlated^2^, enabling us to distinguish molecular pathways that are involved in one or the other process of protein aggregation. While genome-wide association studies (GWAS) have unequivocally pointed to the innate immune system and particularly myeloid cells as major contributors to AD pathophysiology^3–5^, our current understanding of the mechanistic involvement of microglia and infiltrating macrophages in human AD pathology is rudimentary. Much of what shaped our understanding of the role of innate immune cells in AD pathogenesis is based on studies performed in animal models of AD which imperfectly capture the many different aspects of the human disease^6^. In addition, significant differences have been reported between mouse and human innate immune responses in aging^7^ and AD^8^. Human genetic, translational, and imaging studies have implicated myeloid cells in all aspects of AD, from the asymptomatic phase of amyloid accumulation^9,10^ to the progression of dementia^11,12^, but there is little clarity on how disparate observations can be assembled into a coherent picture. Therefore, to identify the different components of human myeloid responses that exist in the neocortex and to examine how each component contributes to the continuum of AD-related pathological and clinical traits in the aging brain, we evaluated a recently derived Molecular Network Map of the aging human frontal cortex using tissue level RNA sequence (RNA-seq) data obtained from the frozen dorsolateral prefrontal cortex of participants in two prospective studies of cognitive aging, the Religious Order Study (ROS) and the Memory Aging Project (MAP)^13–15^. From the RNA-seq data, we derive groups of co-expressed genes—that we term “modules”—and build the network from these modules as well as outcome measures available from each of the 540 participants that were profiled. As these individuals are non-demented at study entry, they represent a sampling of the older, aging population. At the time of death, they display a full spectrum of clinicopathologic features related to AD that are found in older people including cognitive decline, dementia, extracellular β-amyloid deposition, hyperphosphorylation of Tau, and microglial activation^13,16^.

Here, using our recently established gene expression signature of aged human microglia (HuMi_Aged geneset)^17^, we identify five microglia-related modules of co-expressed cortical genes that capture different transcriptional programs of microglia. We focus on dissecting the role of these modules in AD; in mapping the conditional relationship between modules and cognitive and neuropathologic outcomes, we focus on the identification and histologic validation of a microglial module that contributes to the accumulation of Tau pathology. Two other modules relate to β-amyloid, and a fourth—enriched for AD susceptibility genes—appears to be primarily related to aging. Thus, we provide an initial immune network perspective of the divergent sets of co-expressed genes that govern microglial identity in the aging human brain and identify the relationship of these components to specific pathologies and clinical symptoms.

## Materials and methods

### ROSMAP cohort

The subjects profiled in this study are participants in one of two prospective cohort studies of aging, the Religious Orders Study (ROS)^13^ and the Memory and Aging Project (MAP)^14^ which are designed to be merged for joint analyses. These studies enroll non-demented individuals and include detailed, annual antemortem characterization of each subject’s cognitive status as well as prospective brain collection and a structured neuropathologic examination at the time of death. The study design of ROS and MAP yields an autopsy sample that includes a range of syndromic diagnoses and neuropathologic findings that are common in the older population. All brain autopsies, experiments, and data analysis were done in compliance with protocols approved by the Partners Human Research Committee or the Rush University Institutional Review Board. The subjects in the study have an average age of 88, 61% meet criteria for pathologic AD by NIA-Reagan criteria^18^ and 64% are female.

### Description of RNA-Seq from ROSMAP

RNA was sequenced from the gray matter of dorsal lateral prefrontal cortex (DLPFC) of 542 samples, corresponding to 540 unique brains. These samples were extracted using Qiagen’s miRNeasey mini kit (cat. no. 217004) and the RNase free DNase Set (cat. no. 79254). RNA was quantified using Nanodrop. Quality of RNA was evaluated by the Agilent Bioanalyzer. All samples were chosen to pass two initial quality filters: RNA integrity (RIN) score >5 and quantity threshold of 5 µg (and were selected from a larger set of 724 samples). RNA-Seq library preparation was performed using the strand-specific dUTP method^19^ with poly-A selection^20^. Sequencing was performed on the Illumina HiSeq with 101 bp paired-end reads and achieved coverage of 150 M reads of the first 12 samples. These 12 samples will serve as a deep coverage reference and included 2 males and 2 females of non-impaired, mild cognitive impaired, and Alzheimer’s cases. The remaining samples were sequenced with a target coverage of 50 M reads. The libraries were constructed and pooled according to the RIN scores such that similar RIN scores would be pooled together. Varying RIN scores result in a larger spread of insert sizes during library construction and lead to uneven coverage distribution throughout the pool.

The RNA-Seq data were processed by our parallelized pipeline. This pipeline includes trimming the beginning and ending bases from each read, identifying and trimming adapter sequences from reads, detecting and removing rRNA reads, and aligning reads to reference genome. The non-gapped aligner Bowtie was used to align reads to the transcriptome reference^21^, and RSEM was used to estimate expression levels for all transcripts^22^. The FPKM values were the outcome of our data RNA-Seq pipeline. Data are available on https://www.synapse.org/#!Synapse:syn3388564.

For normalization, we first applied quantile normalization to the FPKM values and then used the combat algorithm^23^ to remove potential batch effects. Expression levels were quantified for 55,889 unique genes. We placed a threshold for expression, only keeping 13,153 genes with average FPKM greater than one. For the creation of the modules as previously reported^15^, we used linear regression (on log2 expression data) to remove the effect of major biological and technical confounding factors on a per-gene basis. Biological confounding factors include three genotyping PCs (to represent ancestry), age at death, and sex. Technical confounding factors include RIN, number of ribosomal bases, number of aligned reads, study index (ROS or MAP), and postmortem interval. For the module, pathological association analysis and comparisons between microglia profiles and bulk expression, GC, and length bias effects were first removed using a smoothed trimmed means of M-values before genes were averaged within each module.

### Description of Alzheimer’s disease-related traits

#### Β-amyloid and Tau

To quantify β-amyloid and tau levels present in the brain, tissue was dissected from eight regions of the brain: the hippocampus, entorhinal cortex, anterior cingulate cortex, midfrontal cortex, superior frontal cortex, inferior temporal cortex, angular gyrus, and calcarine cortex. In total, 20-µm sections from each region were stained with antibodies to the β-amyloid beta protein and the tau protein, and quantified with image analysis and stereology, as previously described^2,14,16,24^. Briefly, β-amyloid was labeled with an N-terminus-directed monoclonal antibody (10D5; Elan, Dublin, Ireland; 1:1000). Immunohistochemistry was performed using diaminobenzidine as the reporter, with 2.5% nickel sulfate to enhance immunoreaction product contrast. Between 20 and 90, video images of stained sections were sampled and processed to determine the average percent area positive for β-amyloid (Supplementary Fig. 1a). PHFtau was labeled with an antibody specific for phosphorylated tau (AT8; Innogenetics, San Ramon, CA; 1:1000). Between 120 and 700, grid interactions were sampled and processed, using the stereological mapping station, to determine the average density (per mm^2^) of PHFtau tangles (Supplementary Fig. 1b). The scores across the eight regions were averaged, for β-amyloid and tau separately, to create a single summary measure for each protein. To create approximately normal distributions and facilitate statistical comparisons, we analyzed the square root of these two summary measures.

#### Neuritic plaques, neurofibrillary tangles, and diffuse plaques

Neuritic plaque burden (Supplementary Fig. 1c) and Neurofibrillary tangle burden (Supplementary Fig. 1d) and diffuse plaque burden (Supplementary Fig. 1e) was determined by microscopic examination of silver-stained slides from five regions: midfrontal cortex, midtemporal cortex, inferior parietal cortex, entorhinal cortex, and hippocampus. The count of each region is scaled by dividing by the corresponding standard deviation. The five scaled regional measures are then averaged to obtain a summary measure for both neuritic plaque and Neurofibrillary tangle burden.

#### Cognitive decline

The ROS and MAP methods of assessing cognition have been extensively summarized in previous publications^2,13,25–27^. Uniform structured clinical evaluations, including a comprehensive cognitive assessment, are administered annually to the ROS and MAP participants. Scores from 17 cognitive performance tests common in both studies were used to obtain a summary measure for global cognition as well as measures for five cognitive domains of episodic memory, visuospatial ability, perceptual speed, semantic memory, and working memory. The summary measure for global cognition is calculated by averaging the standardized scores of the 17 tests, and the summary measure for each domain is calculated similarly by averaging the standardized scores of the tests specific to that domain. To obtain a measurement of cognitive decline, the annual global cognitive scores are modeled longitudinally with a mixed-effects model, adjusting for age, sex and education, providing person-specific random slopes of decline. The random slope of each subject captures the individual rate of cognitive decline after adjusting for age, sex, and education. Further details of the statistical methodology have been previously described^28^.

### Microglia morphology

Microglia morphologies had previously been measured with immunohistochemistry for 105 ROSMAP subjects in our study^29^. Immunohistochemistry for microglia was performed using an Automated Leica Bond immunostainer (Leica Microsystems Inc., Bannockburn, IL) and anti-human HLA-DP, DQ, DR antibodies (clone CR3/43; DakoCytomation, Carpinteria CA; 1:100) using standard Bond epitope retrieval and detection. An investigator blinded to the clinical and pathologic data, outlined the cortical gray matter region of interest on each slide using a Microbrightfield Stereology System. The Stereo Investigator 8.0 software program was used to place a 1000 × 750-μm sampling grid over the region and the program was engaged to sample 4.0% of the region with a 200 × 150-μm counting frame at ×400 magnification at interval grid intersection points. Using separate tags for stages 1, 2, and 3 microglia, the operator marked the microglia at each intersection point. These counts were then upweighted by the stereology software to estimate a total number of microglia (stages 1, 2, and 3) in the defined area. This approach relies on the fact that different stages of microglia activation from least (stage 1) to most (stage 3) activated can be defined based on their cellular morphology; when microglia become activated, their long fine processes contract and thicken and the cell body adopts a larger more rounded cellular conformation.

### Activated microglia validation

For six subjects from the clinical core at The Rush Alzheimer’s Disease Center, 6-μm sections of formalin-fixed paraffin-embedded tissue from the DLPFC were used to stain *TMEM119* (Sigma Aldrich) and *VASP* (Santa Cruz Biotech). The sections were blocked with a blocking medium (8% of horse serum and 3% of BSA) and incubated overnight at 4 °C with primary antibodies. Sections were washed with PBS and incubated with fluorochrome-conjugated secondary antibodies (Thermo Fisher) and coverslipped with an anti-fading reagent containing Dapi (P36931, Life technology). Photomicrographs were captured at X20 magnification using Zeiss Axio Observer.Z1 fluorescence microscope and exported to Image J imaging software (NIH, Maryland, USA). For each subject, 30 images were taken in a zigzag sequence along the cortical ribbon to ensure that all cortical layers were represented in the quantification. This analysis was repeated for eight subjects from New York Brain Bank at Columbia University where 10-μm sections of frozen tissue from the DLPFC were used to co-stain *CD45* (Fisher) with *TRABD* (Sigma) or *ACADVL* (Sigma). Phosphorylated tau protein (pTau) (AT8; Fisher) were stained in sister sections. After fixation with methanol or ethanol for 15 min at −20 °C, tissues were blocked with blocking medium for one hour then incubated with primary antibodies overnight at 4 °C. After washing with PBS, tissues are incubated with fluorochrome-conjugated secondary antibodies and mounted with mounting media containing *DAPI*. Between sister sections, images from the same region have been captured with the same approaches as previously described. Levels of pTau were measured as the proportion (%) of the stained area related to the total are of the images.

### Definition of microglia genes

As previously reported^17^, an aged human microglia signature (HuMi_Aged geneset) of 1030 microglia-enriched genes were defined by comparing the expression of genes in DLPFC isolated microglia to their expression level in the bulk DLPFC tissue. The identified genes had fourfold larger expression in the microglia samples relative to the tissue level samples and average FPKM greater than one in the microglia samples.

### Definition of modules from ROSMAP

As previously reported^15^, the SpeakEasy algorithm^30^ was used to derive gene modules from normalized gene expression data. Consensus clustering results from 100 initializations of the SE algorithm yielded 257 modules, 47 of which contained at least 20 gene members (meaning modules are assigned for 98% of genes) and were examined in downstream analyses. Pseudo-expression values for each module were calculated by taking the mean expression level of all genes assigned to that module after the expression data have been standardized for each gene. The gene modules that we used in this paper are the same as those defined in our prior manuscript^15^ which focused on discovering driver genes relating to module 109.

### Modules enriched for microglia genes

Hypergeometric tests were used to test whether modules contained more microglia-related genes than expected by chance. All 13,153 genes that had an FPKM value greater than 1 in the DLPFC ROSMAP data were used as the background for the tests. The HuMI_Aged geneset was used as the primary reference for these analyses. Reference sets derived from two other manuscripts were also used for validation^31,32^. For both datasets, gene counts were TMM normalized and voom^33^ was used to detect microglia-specific genes relative to all other cell types using a Bonferroni adjusted *P* value of 0.05. Our isolated microglia profiles were also compared to our reference RNA-seq profiles of human iPSC-derived neurons and primary human astrocytes (https://www.synapse.org/#!Synapse:syn2580853/wiki/409844) to identify genes whose expression was enriched in microglia, astrocytes or neurons. The microglia-enriched genes were genes that had at least a fourfold increase in expression relative to both the astrocyte profile and the neuron profile. The astrocyte and neuron enriched genes were defined in the same way. We also confirmed cell-type enrichment using cell-type-specific profiles from the mouse cortex tissue^34^ dataset. Using these cell-type-specific gene sets, we then explored the relative expression of the genes within each module in the microglia-astrocyte-neuron gene expression space using ternary plots.

### Pathway analysis of modules

Hypergeometric tests were used to test which Gene Ontology biological process demonstrated an excess in overlap, over what is expected by chance, with each of the five microglial modules^35^. Only biological processes with more than 20 annotated genes or less than 500 genes were tested. Only genes that were assigned to a module and in at least one biological process were included in the hypergeometric tests.

### Transcription factor target analysis of modules

Hypergeometric tests were used to test if the targets of any transcription factors demonstrated an excess in overlap, over what is expected by chance, with each of the five microglial modules. Transcription factor target genes were downloaded from MSigDB from the C3:regulatory target gene sets:TFT: transcription factor targets. Only genes that were assigned to a module and targeted by at least one transcription factor were included in the hypergeometric tests.

### Enrichment of Alzheimer’s disease susceptibility genes

Hypergeometric tests were used to test whether modules contained more genes associated with Alzheimer’s disease than expected by chance. A gene was said to be associated with Alzheimer’s disease if it had at least one probe that was 50 KB up or downstream of the gene that was nominally significant (*P* < 0.05) in IGAP^36^. Only genes that were assigned to a module and contained at least one IGAP probe were included in the tests. The INRICH^37^ GUI v1.0 was also used with default settings to perform enrichment analysis.

### Associations with Alzheimer’s-related traits

#### Testing association with traits

Linear regression analysis was used to associate either single gene expression or average module expression to neuropathologic variables and cognitive decline for all 540 ROSMAP participants. These included the rate of cognitive decline (cogDec) and the square root of the numbers of neuritic plaques (NP), neurofibrillary tangles (NFT), the amount of β-amyloid or tau and. All the associations were adjusted for age, sex, study (ROS or MAP), RIN, and postmortem interval (PMI). A similar analysis was performed including the average expression of neuronal (m21), astrocyte (m107), oligodendrocytes (m110), and endothelial cell (m112) modules in the model to account for major shifts in cell-type proportions.

#### Replication of association in Zhang et al.

We replicated the association of the immune modules with Alzheimer’s disease pathology in an independent microarray gene expression data from the cortex (DLPFC) from a previous study by Zhang et al.^4^. Data were downloaded from GEO with accession number GSE44772. The expression data were quantile normalized. For each module, we created a meta-feature consisting of the average standardized expression of all genes within the module. Linear models including age, sex, postmortem interval, pH, RIN, and the batch was then used to test for association of each module with a diagnosis of Alzheimer’s disease.

#### Replication of association in Allen et al.

We replicated the association of the immune modules with Alzheimer’s disease pathology in an independent microarray gene expression data from the cortex (DLPFC) from a previous study by Allen et al.^38^. Data were downloaded from https://www.synapse.org/#!Synapse:syn5550404. We TMM normalized the expression data. For each module, we created a meta-feature consisting of the average standardized expression of all genes within the module. Linear models including sex, age, and RIN as covariates were then used to test for association of each module with a diagnosis of Alzheimer’s disease.

#### Partial correlation analysis

Partial correlations calculate with the glasso package^39^ in R were used to further disentangle the highly correlated modules and traits. A partial correlation between a trait and a module is the correlation between the trait and module after accounting for the behavior of all other traits and modules. RIN and PMI were included in the partial correlation matrix but removed for visualization. The graphical lasso^39^ was used to set small partial correlations to zero and was tuned using repeated tenfold cross-validation to estimate the penalty parameter.

### Module 116 association with age in H3K9Ac ChIP-seq data

We tested the association of the immune modules with age in an H3K9Ac ChIP-seq dataset generated on the same ROSMAP cohort. For each module, we created a meta-feature consisting of the average peak heights within 10Kb of all genes within the module. Linear models including sex, postmortem interval, RIN, and batch were then used to test for association of module 116 with age.

### Mediation analysis

Mediation analysis was performed by performing linear regression and including or excluding the variables displayed in each of the relevant figures. If the coefficient between two variables A and B has a small *P* value (*P* < 0.05), which then rises above 0.05 when a third variable C is included in the regression, the relationship between A and B is said to be mediated through C. Mediation analysis was performed on the whole cohort and the ROS and MAP cohorts separately.

### Validation in mouse models of AD

Data for two mouse studies were downloaded from GEO with accession numbers GSE64398^40^ and GSE98969^41^. In both datasets, module expression was calculated by calculating the average expression of genes within each module. Only gene homologs that share the same name in human and mouse were included. Both datasets were only explored visually across time and between conditions^40^ and between cell types^41^.

### Validation of activated microglia association via snRNA-seq

A single nuclei sequencing dataset that was generated from 48 subjects from the ROSMAP cohort^42^ was downloaded from https://www.synapse.org/#!Synapse:syn18485175. The authors provided both the raw gene counts and the results from their cell-type clustering. We transformed the counts into cpm, and for each cell calculated the average cpm expression for each of our gene modules. A t-SNE plot of all cells was generated using the average expression of each of the five microglia modules. Four of the authors’ 20 transcriptionally distinct clusters were enriched with microglia markers. We assessed which cluster of microglia cells had the highest m5 expression using a boxplot. For each subject, we calculated the proportion of cells in each of these four clusters relative to the sum of the four clusters. Robust linear regression, using the robust base package in R, was then used to assess the association between the proportions and tau pathology with sex and PMI as covariates.

### Validation of activated microglia association via microscopy

The images were analyzed with the Broad Institute’s Imaging platform, using CellProfiler and CellProfiler Analyst systems to measure and classify cells according to cell morphology^43^. In short, the system provides a semi-automated approach in which an image is automatically segmented into discrete cells from which summary measures of expression per cell are obtained and used in downstream analyses. For the *VASP* + validation, *TMEM119*+ and *VASP*+ cells were first identified using the default thresholding. Overlap of the positive signals was then considered to identify *TMEM119*+*/VASP*+ cells and the lack of co-localization identified the *TMEM119*+*/VASP−* cells. The level of spread or ramification was characterized by the compactness parameter, the variance of the radial distance of the object’s pixels from the centroid divided by the surface area of the object. This measure was then compared between *TMEM119*+*/VASP*− cells and *TMEM119*+*/VASP*+ cells. A random-effects model accounting for between-subject variability was used to assess the significance of differing compactness between the *TMEM119*+*/VASP*− cells and *TMEM119*+*/VASP*+ cells. A similar analysis was performed for both *TRABD* and *ACADVL*.

## Results

### A molecular network map derived from RNA sequence data

ROS and MAP are two large longitudinal studies of aging that were designed and are managed by the same group of investigators so that their data can be merged in joint analyses^13,44^. Participants are non-demented at study entry, agree to brain donation at the time of death, and are evaluated annually with a battery of 21 neuropsychologic tests. A person-specific slope of cognitive decline is calculated for each participant based on 17 tests that are common to the two studies^13^. The demographic and clinical characteristics of the 540 participants used in these analyses are presented in Supplementary Table 1.

The RNA sequence data used in our analyses come from a previously reported dataset^15^ with an average of 95 million paired-end reads of 101 base pairs per participant. To reduce the dimensionality of the RNA-seq data generated from the DLPFC (Dorsolateral Prefrontal Cortex) of each subject, we previously defined modules of co-expressed genes using the Speakeasy algorithm^30^: there are 47 such modules that contain a minimum of 20 genes and a median of 331 genes. Thus, each module contains a group of genes that have a shared regulatory architecture, and the 47 modules represent the nodes of our network^15^. The properties of our modules were tested extensively and discussed in our prior manuscript. Module definitions are fairly similar when compared to those derived from other methods, such as WCGNA, to derive groups of co-expressed genes^15^.

### Identifying microglia-related modules

In this paper, we take a deeper look at the subset of these modules that capture the role of microglia in Alzheimer’s disease and aging. We identified modules enriched for microglial genes as defined in a new reference RNA-seq dataset derived from live, purified human microglia/macrophages extracted from fresh autopsy samples of DLPFC from 10 ROSMAP participants of advanced age: the HuMi_Aged geneset consists of 1,030 microglia enriched genes that were identified based on a fourfold increase in expression in microglia vs. DLPFC expression^17^.

**Fig. 1:**
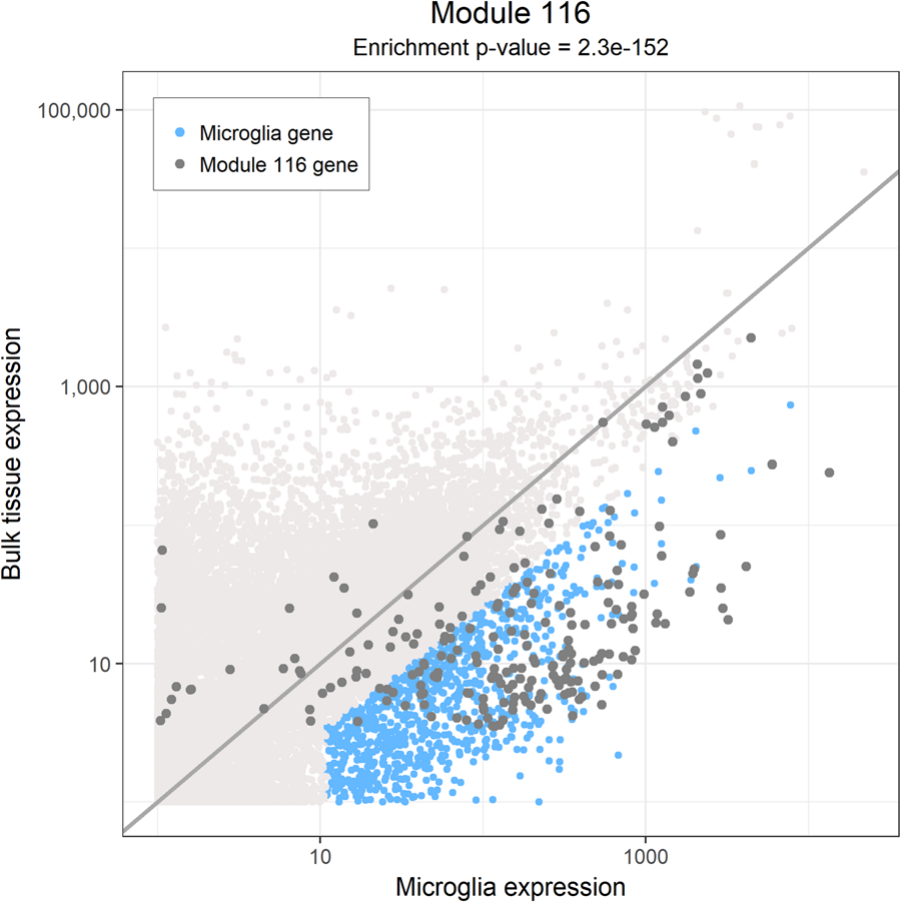
Enrichment analysis identifies m116 as the most microglia-related cortical gene co-expression module. The normalized expression (FPKM) of genes in bulk DLPFC tissue (*Y* axis) are compared to their expression (FPKM) in isolated microglia (*X* axis). A hypergeometric test was used to assess for the extent of enrichment of m116 genes among the 1030 human microglial genes. Each dot represents one gene. Microglia-enriched genes (blue dots) were identified based on a fourfold higher expression in isolated microglia compared to bulk tissue. The microglia-enriched genes are likely involved in processes that are relatively specific to microglia. The genes in m116 are displayed in dark gray. Module 116 genes are more highly expressed in isolated microglia when compared to bulk tissue. FPKM fragments per kilobase of transcript per million mapped reads, DLPFC dorsolateral prefrontal cortex.

Of the 47 modules, five are enriched (*P* < 0.0011, Bonferroni threshold) for the HuMi_Aged geneset: modules m5, m113, m114, m115, and m116 (Table 1, Fig. 1, and Supplementary Fig. 2). Results are similar if we use other human or murine microglial profiles^31,32^ (Supplementary Table 2). To determine whether these enriched modules are specific to microglia, we performed a second set of enrichment analysis using reference RNA-seq profiles of human iPSC-derived neurons and primary human astrocytes (https://www.synapse.org/#!Synapse:syn2580853/wiki/409840). Of the five modules enriched for microglial genes, none are enriched for neuronal genes, but two modules (m113 and m115) are also enriched for astrocyte genes (Supplementary Table 3). The latter modules may relate to immune responses shared with astrocytes, which are well-known to contribute to central nervous system inflammatory responses^45^. By contrast, m5, m114, and m116 appear to be unique to microglia, with m116 displaying, by far, the greatest microglial enrichment: 67% of its 224 genes are present in our list of 1030 microglial genes (Hu_Mi Aged geneset). It contains many well-known myeloid/microglial genes, such as *TREM2* and *TYROBP*. This result is emphasized in a third enrichment analysis of microglial, neuronal, and astrocytic profiles generated from mouse brain^34^ where we observe that m116 is the most cell-type-specific of all tested modules (Supplementary Fig. 3). Given the strength of its enrichment for genes that are reported to be microglial, m116 probably represents a set of genes expressed by all microglia. We note that m116 captures the same set of co-expressed genes that led to the report on the role of *TYROBP* in AD^4^.

**Table 1:** Enrichment of gene co-expression modules for microglia-related genes.

Module	MIcroglia enrichment *P* value	Number of genes in the module	Number of microglia genes in the module	AD genes	Microglia genes associated with cognitive decline	Pathways
M116	2.30E-152	224	149	*TREM2, INPP5D*	*INPP5D, UNC13D, CMTM3, SPP1, TGFBI, RHOG, ACSL1, RPS6KA1*	Positive regulation of immune response, leukocyte activation, adaptive immune response, leukocyte-mediated immunity, response to interferon-gamma
M115	8.60E-17	232	44		*TNFRSF12A, SPATA13, SHMT1, TGIF1, ITGA5*	Inflammatory response, regulation of response to wounding, cellular response to cytokine stimulus, cytokine-mediated signaling pathway, response to external biotic stimulus
M5	1.30E-14	431	58	*BIN1, PVRL2*	*TRABD, PLXNB2, GMIP*	Regulation of JUN kinase activity, regulation of adaptive immune response, leukocyte-mediated immunity, negative regulation of inflammatory response
M113	1.80E-05	313	31	*CLU, SPPL2A, SQSTM1, MPZL1, ETS1*	*ZFP36L1*	Anchoring junction, adherens junction, focal adhesion, cell-substrate adherens junction, cell-substrate junction, wound healing
M114	3.00E-05	276	28		*SCAMP2, FCGRT, POLD1, NINJ1, COLGALT1, IRAK1, NFKBIA, TSPO*	Positive regulation of NF-kappaB transcription factor activity, recycling endosome membrane, cellular response to lipopolysaccharide, cellular response to molecule of bacterial origin, nucleotide-binding oligomerization domain-containing signaling pathway

The five modules are significantly enriched for microglia-related genes (*P* < 0.0011, Bonferroni threshold, hypergeometric test). Also listed are the genes in each module that are close to the AD SNPs in the EBI GWAS catalog, genes that are associated with cognitive decline in the ROSMAP cohort (*P* < 0.006, Benjamini–Hochberg threshold, linear regression) and enriched biological processes.

In our original evaluation of cortical modules^15^, these five microglial modules were all robust: the component genes of each module (see Supplementary Table 4) are (1) co-expressed in an independent, publicly available frontal cortex RNA expression dataset^4^ and (2) correlated in histone 3 lysine 9 acetylation (H3K9Ac, a histone mark of actively transcribed genes) ChIP-seq data from an overlapping set of 669 ROSMAP individuals, suggesting that the co-expression structure is epigenetically driven^15^. To obtain an initial perspective on their function, we annotated each module by identifying both known pathways enriched in each module (Table 1 and Supplementary Table 5) and transcription factors whose binding sites are enriched in the promoters of each module’s genes (Supplementary Table 6). We found m5 to be enriched for genes found in gene sets related to c-JUN N-terminal kinase (JNK) activity, which is consistent with an enrichment of AP1-binding sites in the promoters of m5 genes. This enrichment appears to be specific to m5. Similarly, signatures of phagocytosis are found only in m116, while m113 appears to be specifically enriched for gene sets involved in cell adherence and vascular development. Thus, m113 may reflect a transcriptional program active in perivascular astrocytes and myeloid cells since it is found in both cell types. By contrast, NFκB-related pathways (enriched in m114 and m115) and responses to type I interferons or interferon γ (enriched in m114 and m116) are split between different modules. The transcription factor binding site analysis is consistent with these results, with enrichment in interferon response factor (IRF) binding sites in m116 and in signal transducer and activator of transcription (STAT) binding sites in m115. Notably, the binding site for the SP1/PU.1 transcription factor implicated in AD susceptibility^46^ is enriched in m116 and also, more marginally, in m5. We observe that each module is enriched for unique subsets of transcription factors (Supplementary Fig. 4), suggesting that each module may be maintained by an exclusive set of regulatory mechanisms.

We previously reported that m116 is enriched in AD susceptibility genes (*P* = 0.0002) (Supplementary Table 7), including *TREM2* and *INPP5D* (Table 1)^47^. While not significantly enriched over the background when accounting for the testing of 47 modules using a Bonferroni correction, m5 (*P* = 0.003) also has some evidence of enrichment, with well-validated AD genes such as *BIN1*, being present in m5. These enrichment analyses emphasize the notion that AD susceptibility genes might exert their effect through participating in divergent transcriptional programs of microglia in AD, affecting different aspects of their phenotype and function.

### Modules diverge in their association with AD pathologies

To resolve the relative roles of the different modules in different aspects of AD, we determined their relation to the rich clinicopathologic phenotypes available in ROSMAP participants using a meta-feature calculated for each of the five modules. For our primary univariate analyses, we focused on evaluating two quantitative outcome measures: the amount of amyloid and phosphorylated Tau present in each subject’s brain, as measured by immunofluorescence. These are the two defining pathologic features of AD. Table 2 summarizes all of these results, and we find that, after Bonferroni correction for testing 10 hypotheses (threshold *P* < 0.005), m5 displays a significant association with the burden of Tau (*P* = 0.0028) and a suggestive association with amyloid (*P* = 0.0064), while both m113 (*P* = 0.0043) and m114 (*P* = 0.00015) are associated with amyloid pathology. These two associations are present in both the ROS and MAP subsets of our discovery dataset (Supplementary Table 8). Remarkably, m116, which is the module most strongly enriched for AD susceptibility genes, is positively associated with age but is not strongly associated with cognitive or pathologic measures of AD. Age is the strongest risk factor for AD, so it suggests that AD variants in m116 may primarily have a role in accelerating microglial aging and may not have strong, direct effects on β-amyloid and Tau pathologies or cognitive decline beyond what is accounted for by advancing age.

**Table 2:** *P* values for associations of modules to clinical and pathologic traits.

Module	Activated microglia	Pathologic AD	Clinical AD	Cognitive decline	Neuritic plaques	Diffuse plaques	Neurofi-brillary tangles	Tau	Βeta-amyloid	Sex	Age
M116	0.042 (2.7)	0.16 (0.048)	0.15 (0.049)	0.2 (−0.0094)	0.091 (0.056)	0.61 (0.017)	0.68 (−0.0096)	0.58 (0.048)	**0.016** (0.18)	**0.013** (−0.082)	**0.0049** (1.3)
M115	0.041 (3.1)	0.47 (0.025)	0.16 (0.047)	0.094 (−0.012)	0.099 (0.055)	0.66 (0.015)	0.77 (−0.0068)	0.87 (0.014)	0.022 (0.18)	**0.0044** (−0.094)	0.4 (0.39)
M114	0.02 (2.8)	**0.0069** (0.11)	**0.0045** (0.11)	**0.0035** (−0.025)	**0.00031** (0.14)	0.055 (0.076)	0.24 (0.033)	0.024 (0.23)	**0.00015** (0.34)	**0.00016** (−0.15)	0.5 (0.37)
M113	0.63 (0.64)	0.43 (0.028)	0.026 (0.077)	0.085 (−0.013)	0.1 (0.056)	0.28 (-0.037)	0.75 (0.0075)	0.87 (−0.014)	**0.0043** (0.22)	0.13 (−0.052)	0.11 (0.75)
M5	**0.00042** (4)	**0.005** (0.12)	0.068 (0.077)	**0.014** (−0.022)	**0.0019** (0.13)	**0.00036** (0.15)	0.43 (0.023)	**0.0028** (0.33)	**0.0064** (0.26)	**0.00015** (−0.16)	0.42 (0.46)

Linear regression analysis was used to associate average module expression to neuropathologic variables and cognitive decline for all 540 ROSMAP subjects. Reported are the *P* values with effect sizes in brackets. All the associations were adjusted for age, sex, study (ROS or MAP), RIN, and postmortem interval (PMI). The tests with *P* values <0.016 were significant after a Benjamini–Hochberg correction for multiple hypothesis testing and are highlighted in bold. Only 105 subjects have activated microglia counts.

To address concerns that these associations may be purely driven by changes in proportions of the major cortical cell types, we repeated them after accounting for the proportion of each cell type (Supplementary Table 8); all three associations persist. Further, to test whether the alterations in the expression of m5, m113, or m114 are a late feature of the disease process, we repeated our analyses in the subset of ROSMAP individuals who are cognitively non-impaired at the time of death and find that the associations persist (Supplementary Table 8). This result suggests that these associations are not a feature of terminal AD dementia: they occur earlier in the cascade of events leading to AD, prior to the appearance of cognitive impairment.

To assess whether we can generalize our results, we attempted replication in two additional datasets. We used an existing dataset of frontal cortex data from subjects with AD and subjects without AD and imposed our module definitions on their data^4^; since our quantitative measures of AD neuropathology are not available in these subjects, we attempt replication of the association of modules m5, m113, and m114 with a pathologic diagnosis of AD (which is based on the burden of amyloid and Tau pathologies). The associations of modules m5 (*P* = 1.83 × 10^−5^, *β* = 0.2), m114 (*P* = 1.25 × 10^−5^, *β* = 0.23), and m113 (*P* = 2.7 × 10^−4^, *β* = 0.18) replicate in this repurposed case/control dataset (MSSM1) derived from the same brain region in brain bank samples. Further, we repurposed an RNA-seq dataset from the temporal cortex and the cerebellum of subjects in different diagnostic groups selected from the Mayo Clinic brain bank^38^. Here again, we imposed our module definitions on this third dataset, and we find that m5, m113, and m114 are upregulated in the temporal cortex but not the cerebellum of subjects classified as AD relative to control subjects (Supplementary Fig. 5). Thus, our associations are not limited to the frontal cortex: they are present in another region affected by AD. We also note that this upregulation is not seen in the temporal cortex of subjects classified as having amyloid pathology without cognitive impairment or as having progressive supranuclear palsy, another form of Tauopathy with Parkinsonian features (Supplementary Fig. 5). Given the moderate sample sizes, we cannot definitively say that these two groups do not have upregulation of these microglial modules, but the magnitude of the effect is certainly stronger in AD.

Having replicated the association of our three modules with AD-related traits, we returned to the ROSMAP data to address the specificity of the associations of these modules, we evaluated whether these three modules are associated with Lewy bodies, hippocampal sclerosis, TDP-43, or neurovascular disease. None of these pathologies found in older brains are associated with m5, m113, or m114 (Supplementary Table 8). Thus, we have firmly established the specific association of m5 with Tau pathology as well as m113 and m114 with amyloid pathology.

### Untangling the relationships between age, sex, microglial modules, and AD-related pathologies and traits

**Fig. 2:**
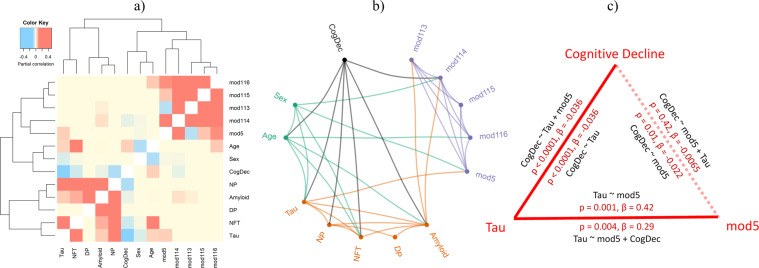
Relationships between gene modules and Alzheimer’s disease traits. The conditional dependencies between the expression level of modules and Alzheimer’s disease traits in the 540 ROSMAP participants are illustrated in (**a**) a heatmap and (**b**) a network diagram. In (**b**), a module or a trait is considered to be a node in the network, and these nodes are connected by lines (edges) if there is evidence that they are associated, after accounting for the behavior of all other modules and traits using partial correlation analysis. **c** Graphical summary of mediation analyses which propose that m5 increased expression precedes the accumulation of Tau which then leads to cognitive decline. All three factors are correlated to one another. On the inner aspect of each edge, we report the *P* value and effect size of the direct association of two traits, which are listed. The outer aspect of each edge presents the results of the model with the third variable included. The dotted edge denotes the fact that the association of m5 and cognitive decline is no longer significant once we adjust for the burden of Tau pathology. This suggests the order of events as being m5→Tau→cognitive decline. All regression models also include RIN and PMI. mod module, CogDec cognitive decline, NP neuritic plaques, DP diffuse plaques, NFT neurofibrillary tangles, RIN RNA integrity number.

AD-related traits are correlated to one another (Supplementary Fig. 6), as are the five microglial modules, and this makes the interpretation of simple univariate analyses challenging, particularly in teasing apart associations with β-amyloid and Tau pathologies. Simply stated through an example, a modest association with β-amyloid in the context of a strong association with Tau could merely reflect the fact that individuals with more Tau pathology tend to have more β-amyloid pathology, leading to a spurious association with β-amyloid for features involved in Tau pathology. However, some molecular features are associated independently with both pathologies. To resolve the most likely set of direct associations between modules and traits, we assessed the conditional dependence between the modules and traits by simultaneously considering all five modules and all pertinent traits to identify the subset of direct module-trait associations that will guide further work. We summarize this statistically rigorous multivariate analysis in Fig. 2a, b, which explores how different modules of the innate immune system may be related to the different components of AD. As expected from our univariate results (Table 2), m114 is still significantly associated with β-amyloid in the multivariate model (the two features are connected by a solid line in Fig. 2b). Likewise, m5 remains significantly associated with Tau. As in the univariate analysis, m113 is also associated with β-amyloid. m113 will not be discussed in detail further due to its high expression in astrocytes which creates ambiguity as to which of the two cell types (or both) may be involved.

Sex has an important but still poorly understood role in AD and microglial function, so we included it explicitly in our model. In our ROSMAP data, sex explains 0.49% of the variance in β-amyloid accumulation and 2.5% of the variance in Tau pathology, which is substantial since APOEε4 explains 2.9% and 0.52% of the variance in these traits, respectively. In Fig. 2a, b, we see that, in our multivariate model that includes age as a covariate, sex influences both m5 (*P* = 0.00015, *β* = −0.16) and m114 (*P* = 0.00016, *β* = −0.15), but not the other three modules. We observe that women have higher levels of both m5 and m114, which is replicated in the repurposed MSSM1 case/control dataset with m5 (*P* = 1.1 × 10^−7^, *β* = −0.39) and m114 (*P* = 1.9 × 10^−9^, *β* = −0.49). To assess whether changes in the expression level of these modules may contribute to the effect of sex on AD and AD endophenotypes, we included the module information in a regression model and found that m114 explains 39% of the variance in amyloid pathology that is explained by sex, and m5 explains 13% of the variance in Tau pathology that is explained by sex (Supplementary Fig. 7a, b). In terms of syndromic diagnoses of AD, m5, and m114 together explain 2.2% of the variance in a pathologic diagnosis of AD and 3.1% of the variance in AD dementia that is explained by sex. Thus, an important proportion of the effect of sex on the accumulation of amyloid appears to involve m114, while m5 plays a significant but smaller role in the effect of sex on Tau pathology.

Advancing age is only associated with higher expression of m116 (*P* = 1.7 × 10^−30^, *β* = 1.3). This result suggests that either the number or proportion of microglia in the neocortex (best captured by m116) is higher with age or that the expression of this set of genes is higher with age. The latter of these two hypotheses seems to be most likely as this m116:age association did not replicate in the H3K9Ac ChIP-seq epigenomic data (*P* = 0.58, *β* = 0.38) or in an evaluation of microglia counts based on histology in relation to m116^10^ (*P* = 0.13, *β* = 19.1), despite its strong effect size in the RNA data. These results suggest that (1) the effect of age is not explained by alterations in chromatin structure, and (2) there is no significant change in the proportion of microglia in the tissue. Thus greater RNA transcription of m116 genes from the same number of microglial cells is the most likely explanation for the association with age. This is consistent with our report that the total number of microglia derived from histological data is not increased in AD^29^. While m116 is not directly related to the pathology measures or to cognitive decline, the overexpression of these genes in microglia of older individuals could contribute to the mechanisms behind aging, which is the single greatest risk factor for AD.

Our multivariate modeling prunes the modest associations (Fig. 2a, b) that may be driven by correlations amongst modules and traits and prioritizes the strongest associations in our data: we clearly see that m113 and m114 are associated with β-amyloid pathology which typically accumulates early in the asymptomatic and early symptomatic phase of AD, and they are not directly associated with tau pathology. On the other hand, m5 is associated with the burden of tau pathology in an β-amyloid-independent manner.

### Mediation analyses

To explore the magnitude of the effect of m5 and m114 on AD traits and to infer the most likely direction of the associations based on our cross-sectional data, we performed mediation analyses using rigorous statistical methods. The results of these analyses must be interpreted cautiously as we do not have a replication dataset large enough to independently confirm our results; nonetheless, these results are useful in prioritizing hypotheses that will be explored in future studies. Cognitive decline, m114, and β-amyloid burden are all associated with one another (Fig. 2), and, after testing each possible mediation model, we find that the best-fitting model is the one in which m114 is upstream of β-amyloid in the sequence of events: in this model, m114 appears to influence cognitive decline through the accumulation of β-amyloid pathology (Supplementary Fig. 7c). A separate analysis suggests that sex may be upstream of m114 (Supplementary Fig. 7a), yielding the following proposed sequence of events: female sex→more m114 expression→more β-amyloid→greater cognitive decline. This is consistent with the observation that the asymptomatic accumulation of β-amyloid pathology occurs for many years before the presentation of symptoms, and our result suggests that one aspect of microglial function (m114) may therefore contribute to β-amyloid accumulation, consistent with functional studies of the *CD33* AD risk allele^10^ which affects the same trait; this effect on amyloid, in turn, contributes to cognitive decline. We note that these results should be interpreted with caution as the results are not powerful enough to be seen consistently in the ROS or MAP cohorts when they are analyzed separately (Supplementary Fig. 7d, e). The separate studies are much smaller than the combined study, so their individual results are less robust. Thus, while the analyses limited to one of the two-component studies contributes to our interpretation of the data, we focus on the results from the larger, combined analysis that considers data from both studies and has the most statistical power.

In parallel, we evaluated the association between m5 and tau. To explore the possible sequence of events among m5 expression, cognitive decline, and tau pathology, we performed a second set of mediation analyses which suggest that the most likely model is that m5 expression precedes tau accumulation and that its effect on cognitive decline may thus be mediated through the accumulation of tau pathology (Fig. 2c). These results are consistent with the previously described putative role of microglia in a mouse model of tau pathology^48^. When we add m5 to a model assessing the effect of sex on Tau burden, we see that the effect of sex is diminished by 13% (Supplementary Fig. 7b), suggesting that m5 may mediate part of the effect of sex on tau accumulation since sex determination occurs before the accumulation of late-life pathologies. However, we highlight that m5 remains significantly associated with Tau pathology after accounting for the effect of sex (*P* = 0.0013, *β* = 0.36) (Supplementary Fig. 7b); thus, the effect of m5 on Tau pathology appears to be influenced both by sex and by other, as yet unknown, factors.

### m5 and m114 in mouse models of AD

While we appreciate that murine models that mimic aspects of amyloid and tau pathology have limited relevance to human disease^6^, we repurposed gene expression data from several mouse models^40^ to assess whether modules m5 and m114 are present in the mouse and whether they are altered in models of CNS amyloidosis and Tau proteinopathy. As seen in Supplementary Fig. 8, we find that both modules are present in the mouse brain and that they increase in expression in two of the five mouse models as the mice age and pathology accumulates. Specifically, mice expressing frontotemporal dementia associated with Leucine instead of the Proline allele at position 301 of human MAPT demonstrate a large increase in module expression between months 8 and 18, consistent with its accumulation of tau aggregates. Further, while individual amyloidosis-related models—the TAS line expressing an amyloid precursor protein containing two familial AD mutations and the TPM line expressing a presenilin 1 gene containing a familial AD mutation—do not show meaningful change in module expression over time, their combination in the homozygous state (HO-TASTPM) does show an elevation of both modules that is apparent at 8 months and enhanced at 18 months as pathology accumulates. However, these mouse models also display similar changes in the other microglial modules (m113, m115, and m116), suggesting that these models have an overall, generalized activation of microglial transcriptional programs that do not recapitulate the more discrete changes that are occurring in the human brain. Further, in a single-cell analysis of the 5× FAD mouse model^41^ of amyloid proteinopathy, all five modules are highly expressed in their homeostatic microglia cell cluster (Supplementary Fig. 9). Relative to all other cell types, none of our pathology associated modules (m5, m113, or m114) are most highly expressed in their two murine microglia clusters that are associated with amyloid proteinopathy. Thus, as noted previously by many investigators, this assessment highlights the fundamental (and increasingly recognized^7,8^) differences that exist between mouse and human microglia in the context of aging, proteinopathy models, and human AD pathology.

### Role of m5 in the aging neocortex

**Fig. 3:**
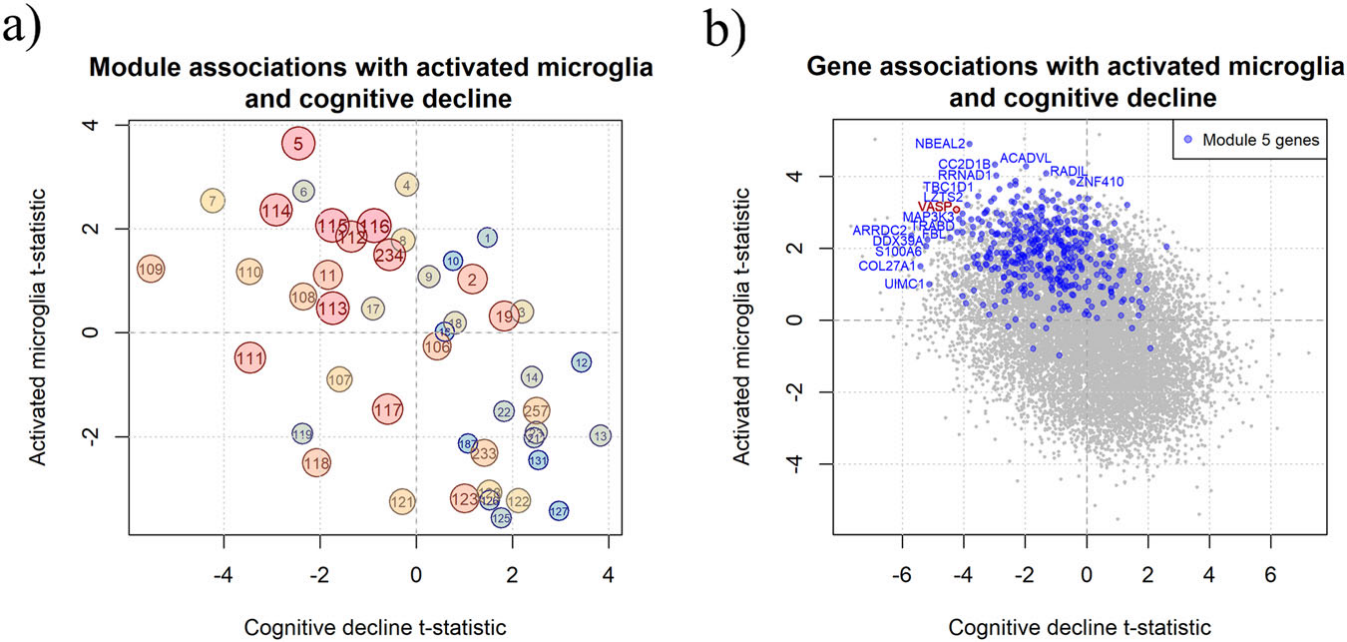
Association of genes and modules with microglia morphology. **a** Scatter plot displaying on the *Y* axis, the t-statistics for associations between module expression in our DLPFC bulk RNA-seq data and the proportion of activated (stage III) microglia and on the *X* axis t-statistics for module expression and cognitive decline. Each circle represents one module, and the number of the module is listed in each circle. m5 is the module most positively associated with the number of stage III microglia. To illustrate the extent of a module’s enrichment for the HuMiAged microglia signature genes, we have both colored the modules (red being more enriched, blue less enriched) and made the size of the circle proportional to the enrichment (the larger circles such as m116 are more enriched for microglial genes). The t-statistics are derived from linear models adjusted for age, sex, study (ROS or MAP), RNA integrity number (RIN), and postmortem interval. **b** Scatter plot displaying on the *Y* axis, the t-statistics for the association of a gene’s expression level and the proportion of activated (stage III) microglia and on the *X* axis t-statistics for the association of the gene’s expression level and cognitive decline. A subset of genes in module 5 which has extreme results are labeled. All genes belonging to module 5 are in blue with VASP highlighted in red. The t-statistics are derived from linear models adjusted for age, sex, study (ROS or MAP), RIN, and postmortem interval.

Since m5 is distinct from m116 in the correlation structure of our RNA-seq data but still very much enriched for genes found in aged human microglia, we hypothesized that it may capture a subset of microglia with a particular function. We, therefore, turned to a phenotype that we had previously captured in our subjects^10^: the proportion of microglia with an activated stage III morphology based on immunohistochemical studies (Supplementary Fig. 10). This neuropathologic measure is available in 104 subjects who also have RNA-seq data. We observe a strong association between the expression of m5 and the proportion of microglia which are categorized morphologically as stage III, activated microglia (*P* value = 0.00042, *β* = 4, Fig. 3a, Supplementary Fig. 11, and Table 2); in fact, out of all 47 cortical modules, m5 is the one most strongly associated with this trait. Module m5 is thus likely to be a transcriptional program related to microglial activation, as defined by morphology-based neuropathological criteria.

To further investigate whether m5 might be indicative of a distinct transcriptional program specific to a subset of microglia, we turned our attention to a single nucleus sequencing (snRNA-seq) experiment^42^. We downloaded these snRNA-seq data and cell clusters that had been generated from the prefrontal cortex of ROSMAP participants. Focusing on the four distinct microglial clusters that were identified by their Louvain graph clustering algorithm (Supplementary Fig. 12a), we observed that the Mic1 cluster had the highest average level of m5 expression (Supplementary Fig. 12b). Furthermore, the proportion of Mic1 cells relative to all microglia cells had a positive association with tau pathology (*P* = 0.043, *β* = 2.2, Supplementary Fig. 12c). These observations provide evidence that there may be a distinct subset of microglia enriched for m5 genes and that the frequency of this subset increases in the context of Tau pathology.

To take these analytic results to the next stage and confirm our results in situ, we selected a representative m5 gene, *VASP*, that (1) had antibodies available for immunofluorescence studies and (2) statistically captured the effect of m5 on tau pathology and cognitive decline. At the single gene level, *VASP* RNA expression in cortical tissue is also strongly associated with activated microglial counts and cognitive decline (Table 3 and Fig. 3b), as well as Tau (*P* = 0.0007, *β* = 0.052). Our modeling suggests that *VASP* should be expressed in microglia and should be expressed at a higher level in microglia that have a morphology consistent with activation according to standard neuropathologic assessment (i.e., have a more globular, stage III morphology).

**Table 3:** Top ten genes in module 5 that are most associated with a combined score for association with activated microglia and cognitive decline.

Gene	Microglia expression	Bulk expression	*P* value activated microglia	*P* value cognitive decline	Combined score
*NBEAL2*	6.1	9.2	3.90E-06	0.00015	8.72
*ARRDC2*	61.5	25.5	0.021	1.90E-08	8.06
*DDX39A*	99.4	28.7	0.027	3.10E-07	7.43
*CC2D1B*	15.4	20	3.60E-05	0.0029	7.32
*VASP*	23.6	12.2	0.0029	2.60E-05	7.3
*S100A6*	14.5	283	0.042	2.60E-07	7.28
*LZTS2*	16.2	34.4	0.0018	0.00011	7.09
*MAP3K3*	22.1	25.2	0.0038	5.90E-05	7.02
*TRABD*	78	13.1	0.0059	3.70E-05	6.98
*TBC1D1*	33.3	78.7	0.00085	0.00046	6.97

Linear regression analysis was used to associate gene expression to activated microglia for 105 ROSMAP subjects and cognitive decline for the genes in module 5 for all 540 ROSMAP subjects. All the associations were adjusted for age, sex, study (ROS or MAP), RIN, and postmortem interval (PMI). Reported are the average expression in microglia and bulk tissue (FPKM), *P* value of the associations and the score that was used to combine the *P* values and rank the genes.

**Fig. 4:**
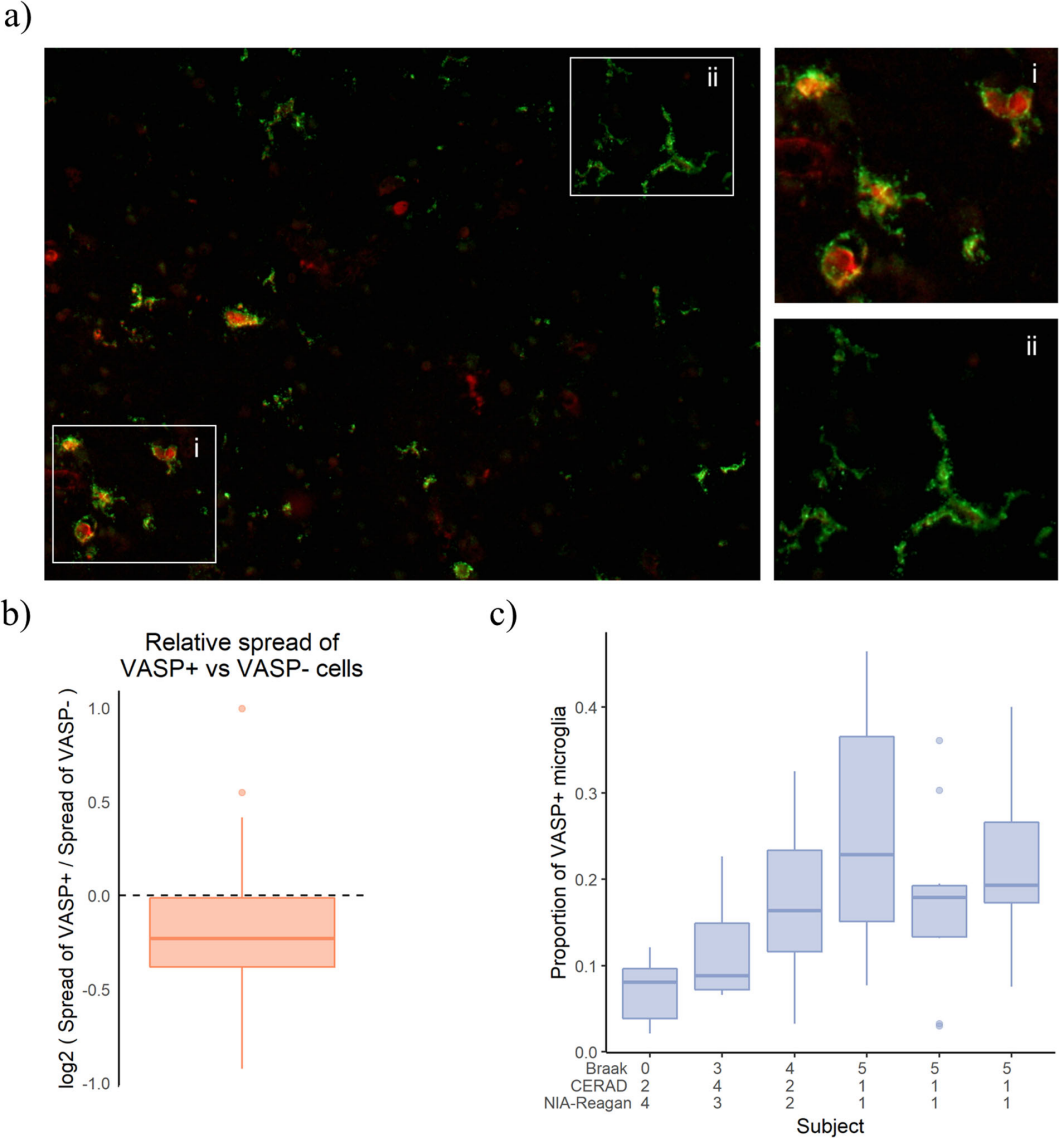
Immunofluorescence reveals VASP + TMEM119+ cells have activated morphology. **a** Representative micrograph showing co-immunostaining of VASP (red) and TMEM119 (green) in DLPFC. Please note the less ramified morphology of TMEM119+/VASP+ cells (inset “i”) when compared to TMEM119+/VASP− cells (inset “ii”). **b** Comparison of cellular morphology (“compactness” is used to quantify the level spread or ramification) between VASP+ and VASP− microglia cells (TMEM119+) for each of the six tested subjects that were analyzed. Overall, a random-effects model integrating data from all six subjects and a total of 4,158 TMEM119+ cells measured demonstrates that VASP-positive microglia are significantly less ramified when compared to VASP-negative microglia (*P* = 3 × 10^−8^, *β* = −0.26). **c** The proportion of TMEM119+/VASP+ to TMEM119+/VASP− relative to Braak, CERAD, and NIA-Reagan scores in six subjects. Linear regression provides evidence that the proportion of VASP-positive microglia increases with Braak score (*P* = 0.012, *β* = −26.17). DLPFC dorsolateral prefrontal cortex, VASP vasodilator-stimulated phosphoprotein, TMEM119 transmembrane protein 119, a pan-microglia marker.

While *VASP* may be a representative gene for immune module m5, it has not been explicitly reported to be expressed in microglia previously. We first demonstrated that *VASP* is expressed in cells labeled with *TMEM119*, a protein proposed as a pan-microglial marker not expressed on infiltrating macrophages^49^ (Fig. 4a). Further, only a subset of *TMEM119*+ microglia is *VASP*+. While *VASP* is expressed in human microglia^34^, it is also found to be expressed in fetal human astrocytes and to a much lesser extent in other cells of the CNS parenchyma. In our histological data, we find it to be present in GFAP+ astrocytes as well as TMEM119+ microglia.

To evaluate whether, m5 and its single gene proxy VASP are truly markers of activated microglia, we used automated image analysis^43^ to assess whether the subset of *TMEM119*+ cells that were also VASP+ had an activated morphology. To minimize bias, we used CellProfiler to identify individual *TMEM119*+ cells and then captured (1) the level of *VASP* expression in each cell and (2) several different morphologic features of each cell. Surveying the 4158 *TMEM119*+ cells captured from cortical tissue sections of six subjects obtained from Rush Alzheimer’s Disease Center, we find that *TMEM119* + *VASP* + microglia are less ramified than *TMEM119* + *VASP−* cells (*P* = 3 × 10^-8^, *β* = −0.26) (Fig. 4b). This association with morphology confirms that *VASP* may be an appropriate surrogate marker for m5 since we found a strong positive association between m5 expression and stage III-activated microglia (Fig. 3a and Supplementary Fig. 11). We examined the relation of VASP expression to tau pathology by (1) showing that, in these six subjects, the proportion of VASP + microglia cells is greater with increasing Braak score (*P* = 0.012, *β* = −26.17) (Fig. 4c), a measure of Tau propagation, and (2) measuring the amount of tau immunofluorescence in a sister section of the section used to measure VASP expression, which shows that the proportion of VASP + cells increases with increasing Tau burden (Supplementary Fig. 13). To further validate these results, we selected two additional m5 marker genes, *TRABD* and *ACADVL*, and we found consistent results when we repeated the VASP analyses with these two other m5 marker genes (Supplementary Fig. 14) in the DLPFC of eight subjects with AD from the New York Brain Bank. Thus, we have robust evidence both that m5 genes are expressed at the protein level in microglial cells that have a more rounded morphology consistent with stage III-activated microglia and that the abundance of microglia expressing m5 genes increases in relation to the local burden of tau pathology. Finally, we can now prioritize VASP, TRABD, and ACADVL for further evaluation as a proxy for the morphological staging of microglia, which is difficult to standardize across individual raters.

## Discussion

We performed a detailed in silico dissection of five cortical transcriptional modules enriched for genes found in aging human microglia and differentiated the role of m116 as a module reflecting microglial aging from those of m113 and m114 that may be involved in promoting the accumulation of β-amyloid and of m5 that captures a morphologically activated microglial state that may contribute to the accumulation of Tau. Thus, we begin to empirically differentiate groups of microglial genes that work together to accomplish specific, distinct functions. This regulatory architecture is present at the chromatin level: our H3K9Ac data both confirm the correlation of genes within RNA-seq-defined modules and validates their association with AD-related traits. It is also reflected by largely-non-overlapping patterns of transcription factor binding site enrichment for each module (Supplementary Table 6).

Importantly, given our large sample size, we can identify different modules involved in β-amyloid and Tau pathology. Our analyses suggest the existence of multiple microglial functional modules active in parallel within the same brain: these transcriptional programs are working in parallel. In the case of m5, we pushed validation efforts further to confirm, in situ, the hypothesis that it reflects the presence of a subset of microglia that are more activated and found in greater numbers as Tau accumulation increases in the tissue, a result consistent with immunohistochemistry-based observations on this cohort^29^. Our data, therefore, implicate microglia in both β-amyloid and Tau accumulation, and the three distinct transcriptional modules (m5, m113, and m114) are all present in the brain of aged individuals. This observation highlights the complexity that we face in developing immunomodulatory therapies for AD: they will have to be tuned to specifically engage or shut down a given transcriptional module while not exacerbating the ongoing perturbation of others. In the case of Tau, we propose the hypothesis that increased expression of m5 by microglia contributes to the accumulation of Tau aggregates; this is the most likely scenario based on our cross-sectional data and robust methods for modeling mediation statistically. However, we cannot determine formal causality with cross-sectional data, and longitudinal studies using appropriate combinations of PET tracers or other biomarkers for Tau and microglial function are needed to validate this putative causal chain. We note that this hypothesis is consistent with the results of our modeling of histological measures of microglial activation^29^ which proposes that morphologically activated microglia contribute to the accumulation of Tau pathology. Thus, transcriptional and histological data converge to suggest that increased prevalence of activated microglia leads to more Tau accumulation, suggesting that downregulation of m5 may be a therapeutic option. The relatively specific enrichment of the JUN kinase pathway in m5 suggests that it may be regulated in a manner that is somewhat distinct from the other four modules and could provide a relatively specific target for drug development.

A surprising result is the lack of strong association between m116 and β-amyloid or Tau pathology: it is contrary to a simple narrative that this module, enriched for AD susceptibility genes from GWAS, exerts its effect primarily through the increased accumulation of the neuropathologies that define AD. β-amyloid and Tau burden only explain some of the variance of cognitive decline and AD dementia^16^, and our m116 results suggest that microglia may be involved in AD through mechanisms that remain elusive today. We clearly demonstrate the presence of an effect of aging on microglia, but this effect does not translate directly into pathology in our analysis. One thing to appreciate is that many AD GWAS often use samples of convenience as control subjects: these subjects are only coarsely characterized. Further, since AD incidence increases with advancing age, the majority of control subjects are likely to develop AD if they live long enough. Thus, the AD GWAS may have uncovered genes that have a strong effect on advancing the age of onset of AD and emerge as susceptibility genes because of the study design. Such an effect would tie in with our results where *TREM2, INPP5D*, and other genes found in m116 are associated primarily with microglial aging^7^.

One possibility is that the different transcriptional modules described here correspond to different microglia subpopulations. Given their sentinel and effector nature, the diversification of microglia phenotypes in specialized niches in the aged brain parenchyma burdened with β-amyloid and Tau deposits is a possible scenario. A longstanding observation is the morphological transformation of parenchymal microglial cells in and around amyloid plaques in AD^50^. Nonetheless, until now the molecular identity of the plaque-associated (ameboid or stage III) activated microglia was obscure. This study takes the first steps to assign, though in an indirect way, a transcriptomic signature to the morphologically defined stage III subpopulation of microglia. VASP, TRABD, and ACADVL may be surrogate markers for stage III microglia.

Nonetheless, our study has certain limitations; foremost amongst these is the fact that our pathologic measures are cross-sectional since they are obtained at autopsy. We therefore cannot comment formally on causality or on the exact sequence of events that is occurring in the living human brain. Our analysis of mouse data from the TAU, TAS, and TPM models suggest that their utility in dissecting human microglial responses to Tau and amyloid aggregation may be limited: they do not recapitulate the observations that we report in the ROSMAP samples and validate in two independent collections of human samples. Further, this neuroimmune network map should be seen as a first draft: microglia represent a minority of the cells found in the human cortex and have a relatively low quantity of mRNA in their cytoplasm. Thus, the level of expression of microglial genes, particularly those present only in a subset of microglia is likely to be underestimated or even absent from the tissue level data (e.g., *CD33*). Further, these microglial modules are likely to contain genes expressed in peripheral monocytes. However, given that monocytes represent such a small minority of the myeloid cells found in the brain, their effect on the cortical transcriptome is likely completely diluted and unlikely to explain the associations that we have uncovered. Second generation maps derived from isolated microglia and transcriptional profiles of single microglia will be essential to better understand the involvement of microglia in the pathophysiology of AD and the complex architecture of microglial subsets in the aged and AD brain.

Overall, we have generated an initial framework on which we and others can assemble additional data from in vivo and in vitro experimental models: when perturbing a particular gene of interest, we have to be cognizant of its membership to a given module of co-expressed genes and to the effect of such perturbations on different transcriptional programs found in microglia that may not be directly measured in an experimental system. The immune system is exquisitely modulated by a complex set of checks and balances in which AD genes such as *CD33*, *TREM1*, and *TREM2* are contributing to regulate the level of activation^9^, and, as seen in other immune functional programs, small differences in the level of receptor engagement or the presence of co-stimulatory molecules can result in dramatically different responses that can sometimes be the opposite of an anticipated response. By refining the set of microglial genes implicated in β-amyloid vs. Tau pathology, we mark a step forward in better targeting drug development programs both by proposing new targets and by defining novel outcome measures that can be used to assess the functional consequences of lead compounds.

## Supplementary information

Supplementary material

Supplementary table 4

Supplementary table 5

Supplementary table 6

Supplementary table 8

## Data Availability

All statistical analysis was performed in R. Code for generating the main figures is available at https://github.com/ellispatrick/microgliaModules.
