# Supplementary material for: A cortical immune network map identifies distinct microglial transcriptional programs associated with β-amyloid and Tau pathologies
Source: Transl Psychiatry. 2021 Jan 14;11:50. doi: 10.1038/s41398-020-01175-9 (PMC7809035; doi:10.1038/s41398-020-01175-9)
Supplement: Supplementary file 1 — Supplementary material [file 41398_2020_1175_MOESM1_ESM.docx]

# **Supplementary material**

## **Supplementary Table 1 – Demographics of participants analyzed in the RNAseq analyses.**

| Study, (ROS, MAP) | (296,245) |
| --- | --- |
| Age, mean (sd) | 88.6 (6.6) |
| Sex, (female, male) | (341,200) |
| PMI, mean (sd) | 7.2 (4.8) |
| RIN, mean (sd) | 7.2 (0.96) |
| NP, mean (sd) | 0.6 (0.47) |
| NFT, mean (sd) | 0.6 (0.34) |
| Pathological AD (not AD, AD) | (220, 321) |

Abbreviations: ROS – Religious Orders Study; MAP – Memory Aging Project; PMI – post mortem interval; RIN – RNA integrity number; NP – neuritic plaques; NFT – neurofibrillary tangles; AD – Alzheimer’s disease.

## **Supplementary Table 2 – Enrichment of gene co-expression modules for microglia related genes.**

| Module | Number of genes in Module |  | (OLAH)  microGlia Enrichment  p-value | (OLAH)  Number of microglia genes in module | (Zhang)  microGlia Enrichment  p-value | (ZHANG)  Number of microglia genes in module | (DARMANIS)  microGlia Enrichment  p-value | (DARMANIS)  Number of microglia genes in module |  |
| --- | --- | --- | --- | --- | --- | --- | --- | --- | --- |
| m116 | 224 |  | 2.30E-152 | 149 | 2.60E-112 | 119 | 1.60E-91 | 150 |  |
| m115 | 232 |  | 8.60E-17 | 44 | 2.00E-05 | 24 | 1.30E-16 | 74 |  |
| m5 | 431 |  | 1.30E-14 | 58 | 1.90E-07 | 40 | 1.10E-02 | 64 |  |
| m113 | 313 |  | 1.80E-05 | 31 | 1.00E-03 | 25 | 1.80E-24 | 105 |  |
| m114 | 276 |  | 3.00E-05 | 28 | 5.40E-01 | 11 | 2.60E-24 | 52 |  |

Modules are tested for enrichment of microglia genesets from three independent studies using a hypergeometric test. Three microglia signatures are used 1) our own^1^ (Olah); 2) a microglia gene signature from a bulk RNA sequencing dataset on isolated cell types from human brain cancer and epilepsy surgery specimens^2^ (Zhang) and 3) microglia genes from a single-cell sequencing study on surgically removed human temporal lobe epilepsy tissue^3^ (Darmanis). Modules are significantly enriched in genes from a signature if the p-value falls below the adjusted (Bonferroni) threshold of p<0.0011.

## **Supplementary Table 3 – Relative enrichment for microglial, astrocytic and neuronal genes in the five modules identified as enriched in microglial genes.**

To assess whether the five modules are enriched only for microglial genes, a second set of enrichment tests were performed. Isolated microglia were compared to reference RNAseq profiles of human iPSC-derived neurons and primary human astrocytes (https://www.synapse.org/#!Synapse:syn2580853/wiki/409844) to identify genes whose expression was more specific to microglia, astrocytes of neurons. The microglia specific genes were genes that had at least a 4-fold increase in expression relative to both the astrocyte profile and the neuron profile, the astrocyte and neuron specific genes were defined in the same way. Here it can be seen that most of the immune modules still appear to be immune related with the exception of module 113 which appears more astrocyte-like and module 115 which appears to be capturing genes specific to both microglia and astrocytes.

| Module | Microglia | Astrocyte | Neuron |
| --- | --- | --- | --- |
| 116 | 5.40E-110 | 0.93 | 1 |
| 5 | 3.40E-10 | 0.68 | 0.97 |
| 115 | 6.80E-07 | 9.10E-06 | 0.093 |
| 114 | 0.0064 | 0.42 | 0.31 |
| 113 | 0.072 | 4.00E-08 | 0.87 |

## **Supplementary Table 4 – Genes in the five microglial modules and their association to AD traits.**

This table is uploaded as a separate pdf. It includes the 1397 genes that are found in m116, m115, m114, m113 or m5. The table includes information on their average expression in isolated microglia and bulk cortical tissue and on whether they were included in the HuMi_Aged gene signature; it also includes results of their association with activated microglia counts, cognitive decline, tau, amyloid, neuritic plaques (NP) and neurofibrillary tangles (NFT).

## **Supplementary Table 5 – Pathway enrichment.**

The 2633 pathways that were over-represented in either m116, m115, m114, m113 or m5 at a nominal significance threshold of 0.05 are listed in this table.

## **Supplementary Table 6 – Transcription factor binding site enrichment.**

The 140 transcription factors whose target genes were over-represented in either m116, m115, m114, m113 or m5 at a nominal significance threshold of 0.05 are listed in this table.

## **Supplementary Table 7 – Table of the enrichment of AD susceptibility genes in each module.**

All 47 cortical modules are tested, so the Bonferroni-corrected threshold of significance is p<0.0011. Module 116 and module 5 were the top two modules when tested for enrichment of AD susceptibility genes with IINRICH or an over-representation test. Of the 47 modules only module 116 is significant in the over-representation test (OR).

|  | Number Genes in module (INRICH) | Number of genes that were SNPS (INRICH) | p-value (INRICH) | Number Genes in module (OR) | Number of genes that were SNPS (OR) | p-value (OR) |
| --- | --- | --- | --- | --- | --- | --- |
| mod116 | 206 | 11 | 0.0037 | 224 | 12 | 2.00E-04 |
| mod5 | 348 | 15 | 0.013 | 431 | 15 | 0.003 |

## **Supplementary Table 8 – P-values for associations of modules to clinical and pathologic traits in subsets of cohorts.**

Linear regression analysis was used to associate average module expression to neuropathologic variables and cognitive decline for subsets of the 540 ROSMAP subjects. Reported are the p-values with effect sizes in brackets. All the associations were adjusted for age, sex, study (ROS or MAP), RNA Integrity number (RIN) and post-mortem interval (PMI). Cohorts are split by non-demented only, ROS only and MAP only. Also included are associations with TDP43, hippocampal sclerosis, Lewy bodies and cerebral atherosclerosis. Also included are the associations between modules and pathologies after accounting for cell-type modules including m21 (neurons), m107 (astrocytes), m116 (microglia), m110 (oligodendrocytes) and m112 (endothelial cells).

## **Supplementary Figure 1 – Images used for pathological measures**

Representative images for the quantification of AD pathology for the variables **(a)** amyloid, **(b)** tau, **(c)** neuritic plaques (NP), **(d)** neurofibrillary tangles (NFT) and **(e)** diffuse plaques (DP) as described in the online methods.


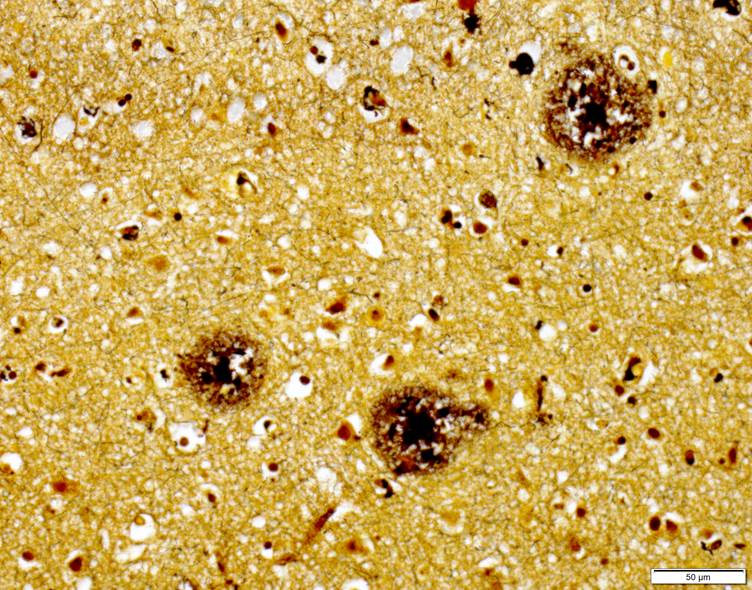

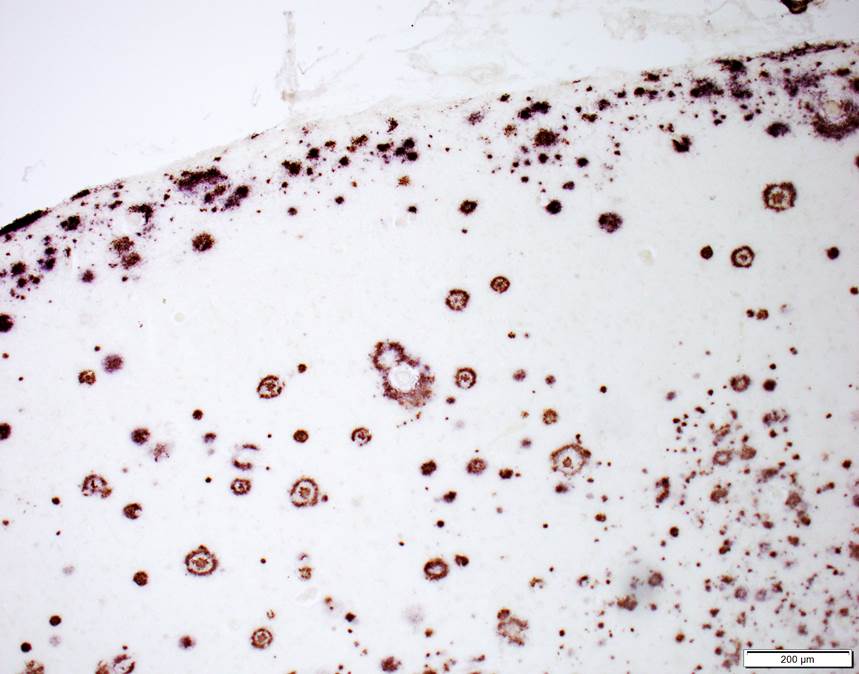

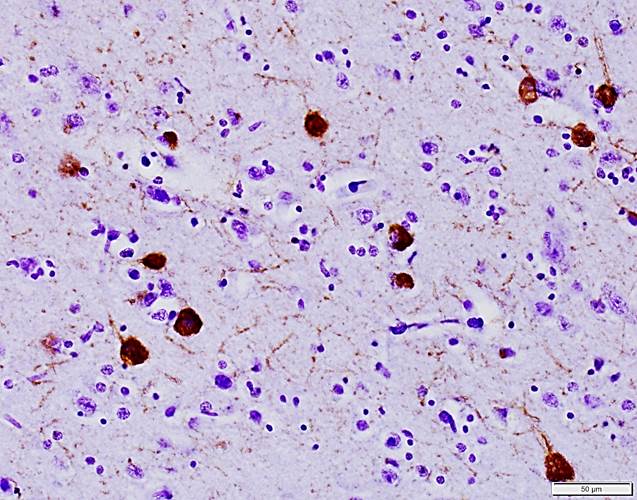

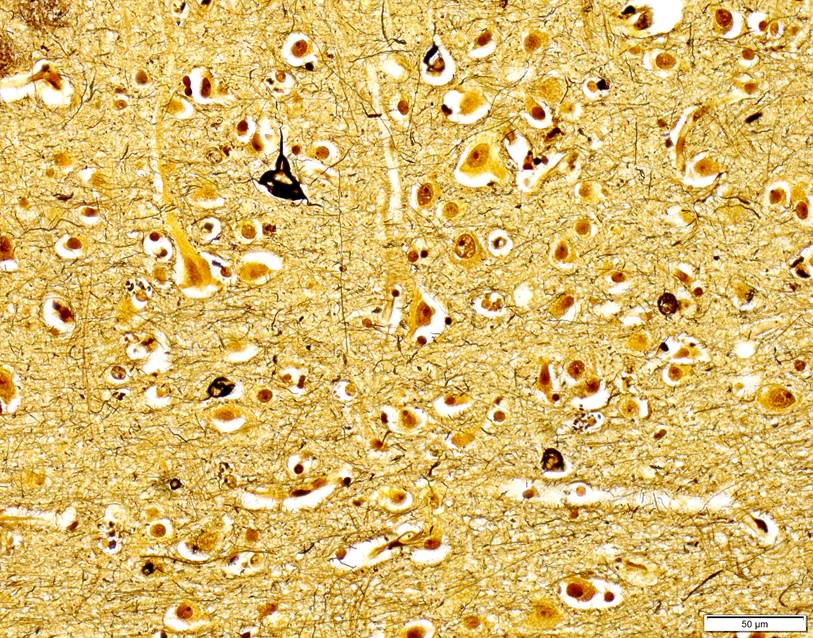

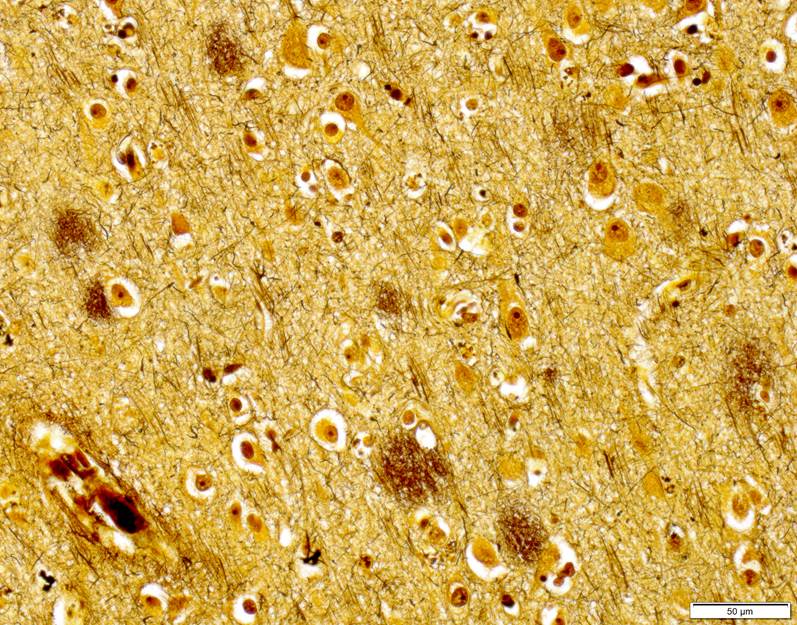


a)

b)

c)

d)

e)

## **Supplementary Figure 2 – Distribution of expression levels for each gene found in each of the 5 modules in microglia vs bulk tissue space.**


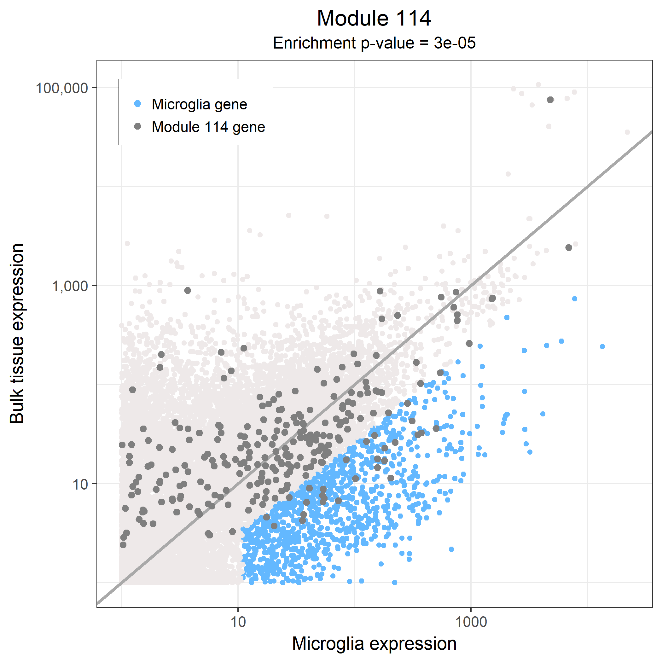

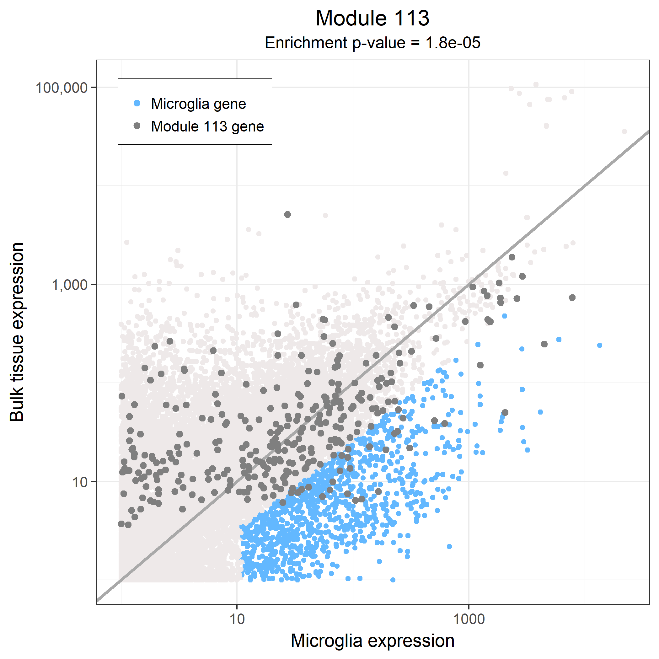

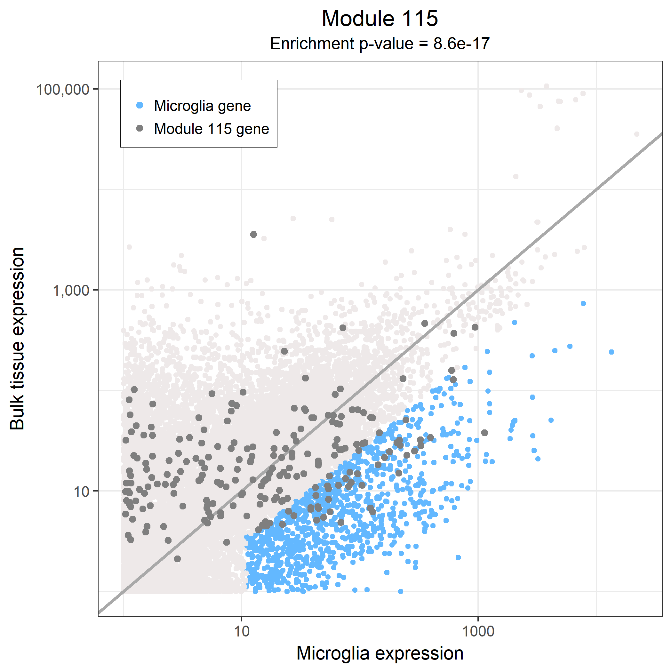

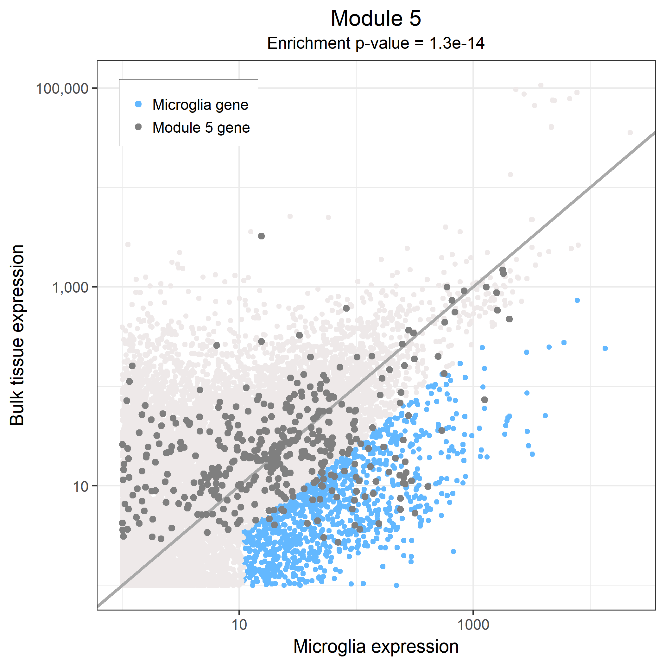
The expression (fragments per kilobase of exon model per million reads mapped, FPKM) of genes in bulk dorsolateral prefrontal cortex (DLPFC) tissue are compared to their expression in isolated microglia. The genes in immune modules 5, 114, 113 or 115 are highlighted in dark gray, and the HuMiAged genes are highlighted in blue. All other measured genes are shown in light gray.

## **Supplementary Figure 3 – Ternary plots showing the relative expression of the immune module genes in a mouse microglia-astrocyte-neuron gene expression space.**

The expression of the genes in our immune modules was compared in microglia, astrocyte and neuron expression profiles isolated from mouse cortex ^4^. The relative expression of each gene in an immune module was plotted in a ternary diagram. Here it can be seen that module 116, 5 and 114 genes all have higher expression in microglia than neurons and astrocytes. The genes in module 113 appear to have higher expression in astrocytes. There appears to be two populations of genes in module 115, one more highly expressed in microglia and one more highly expressed in astrocytes. In the bottom right panel, we plot the distribution of the expression level of each of the modules themselves.


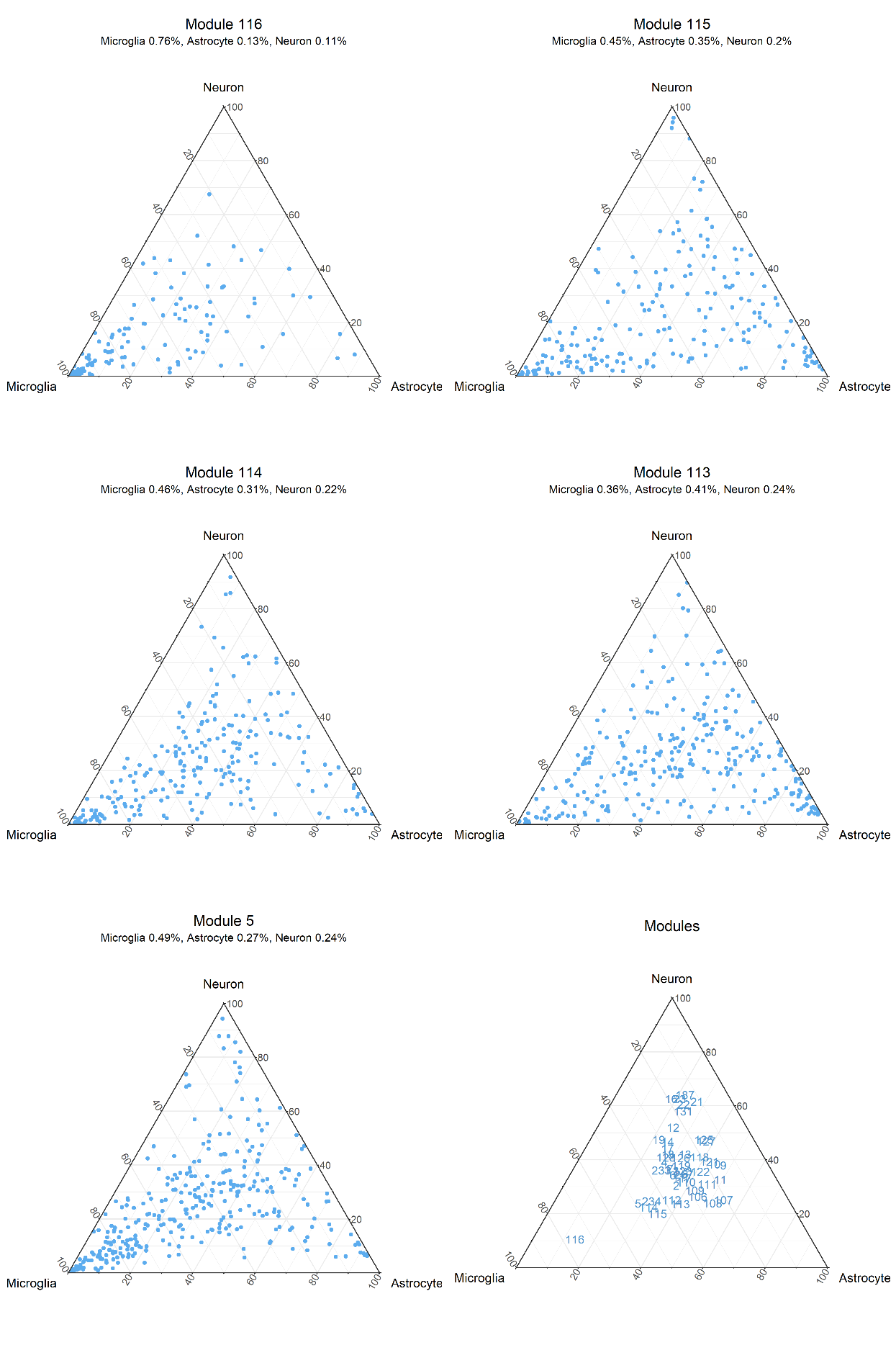


## **Supplementary Figure 4 – Venn diagram of TF genes.**

A Venn diagram illustrating the overlap of the 140 transcription factors whose target genes were over-represented in either m116, m115, m114, m113 or m5 at a nominal significance threshold of 0.05. There is limited overlap amongst the different modules, consistent with the fact that the modules are defined based on gene co-expression patterns.


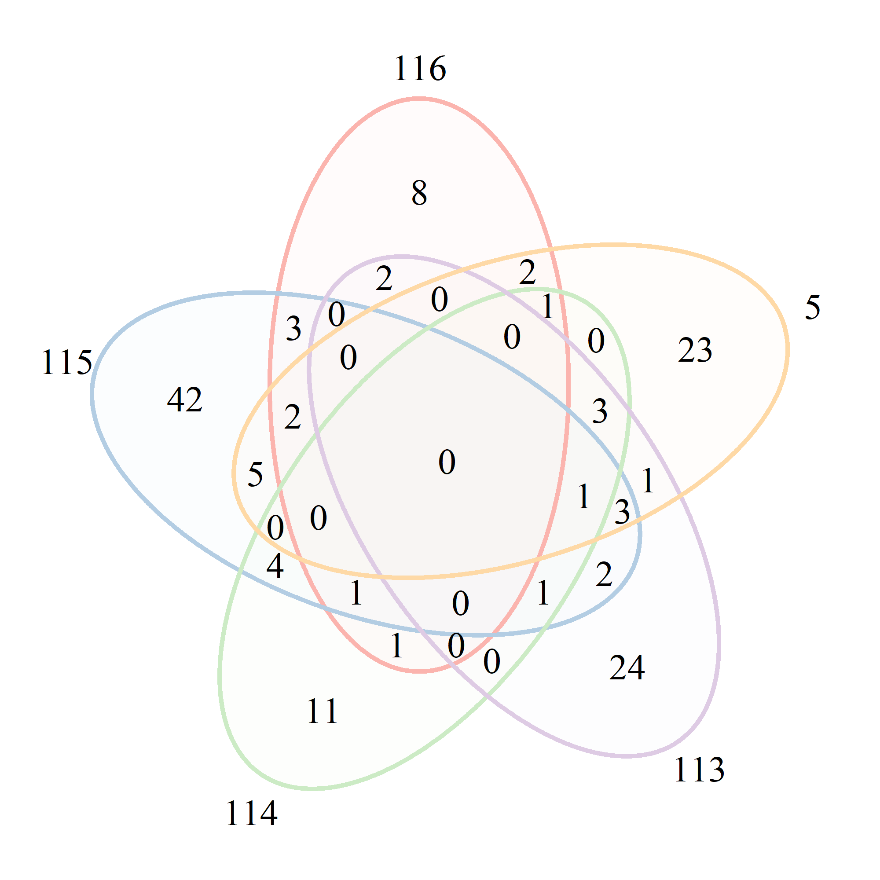


## **Supplementary Figure 5 – Associations of module expression with AD diagnosis in the Mayo samples.**


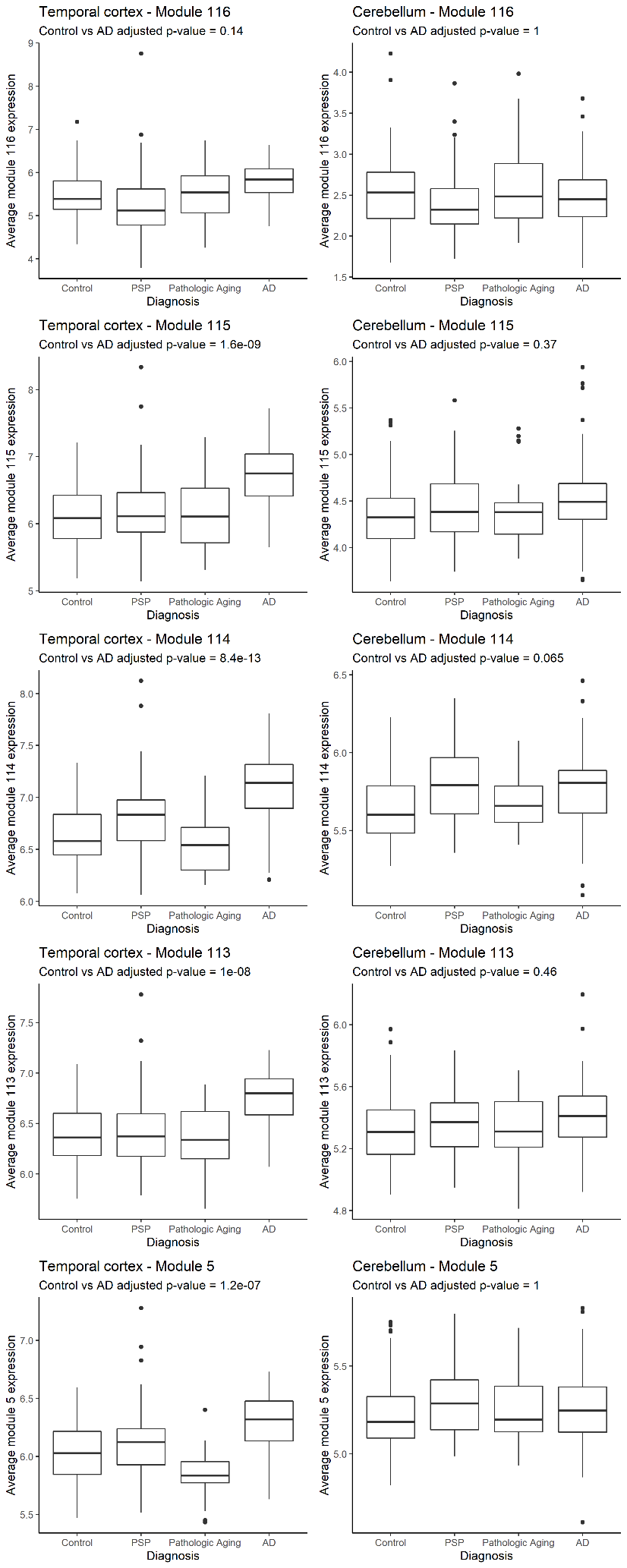
Average module expression from the temporal cortex and the cerebellum of subjects in different diagnostic groups selected from the Mayo Clinic brain bank. Expression values have been adjusted age, sex and RIN. Abbreviation: PSP progressive supranuclear palsy.

## **Supplementary Figure 6 – Correlations of neuropathologic and clinical traits in the ROSMAP subjects used in this study.**

The r value for each correlation is shown in each square and colored using the scale shown to the right of the image. Abbreviations: NFT - neurofibrillary tangles; DP - diffuse amyloid plaques; NP - neuritic amyloid plaque; CogDec - trajectory of cognitive decline.


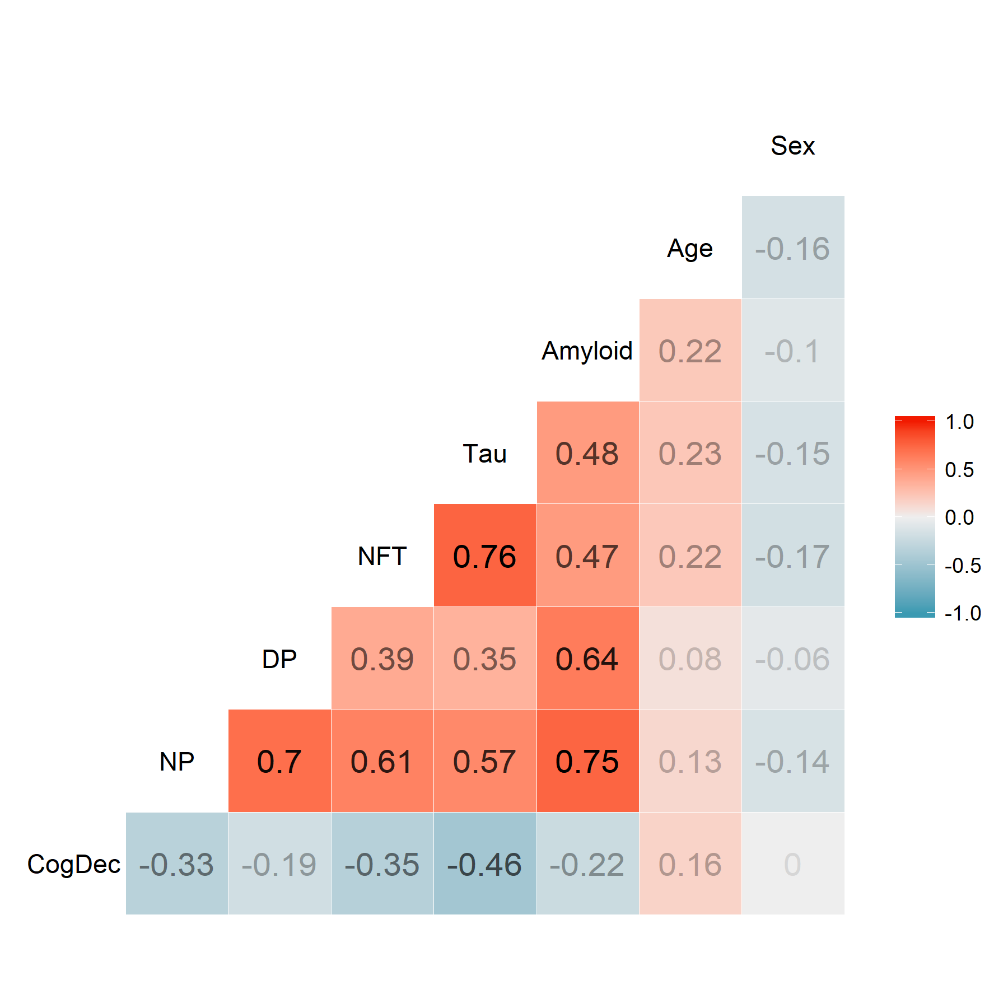


## **Supplementary Figure 7 – Mediation analysis.**


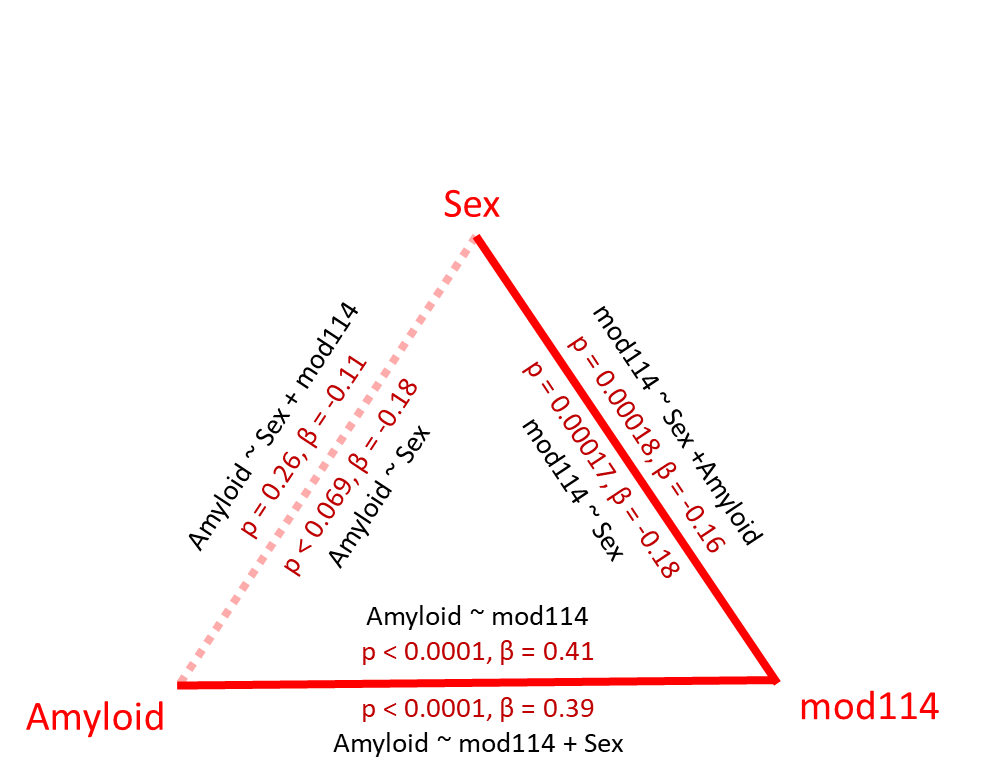

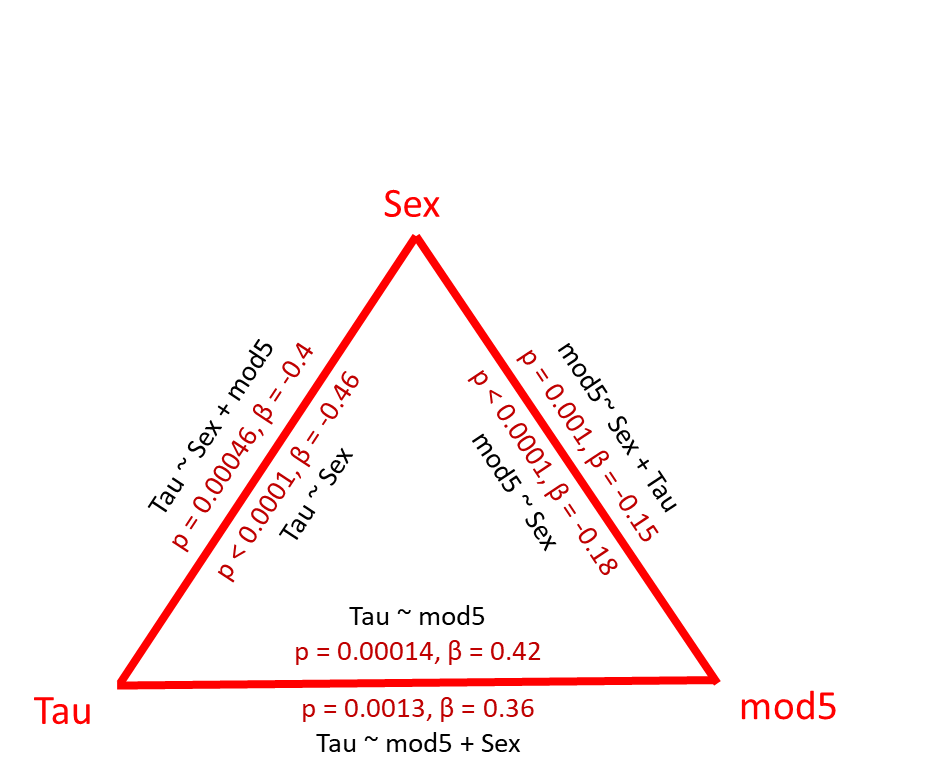
Mediation analysis assessing plausible directions between cognitive decline, pathology, sex and modules. All regression models also include RIN and PMI and are performed either on all samples from ROS and MAP, or each of the cohorts individually.

a) All samples


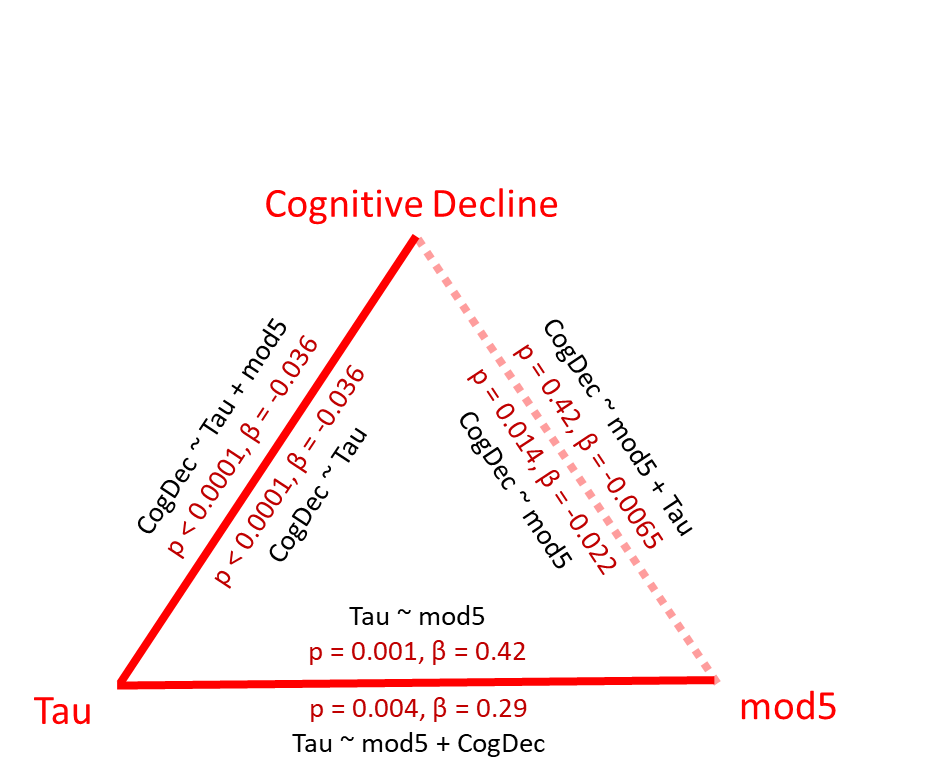

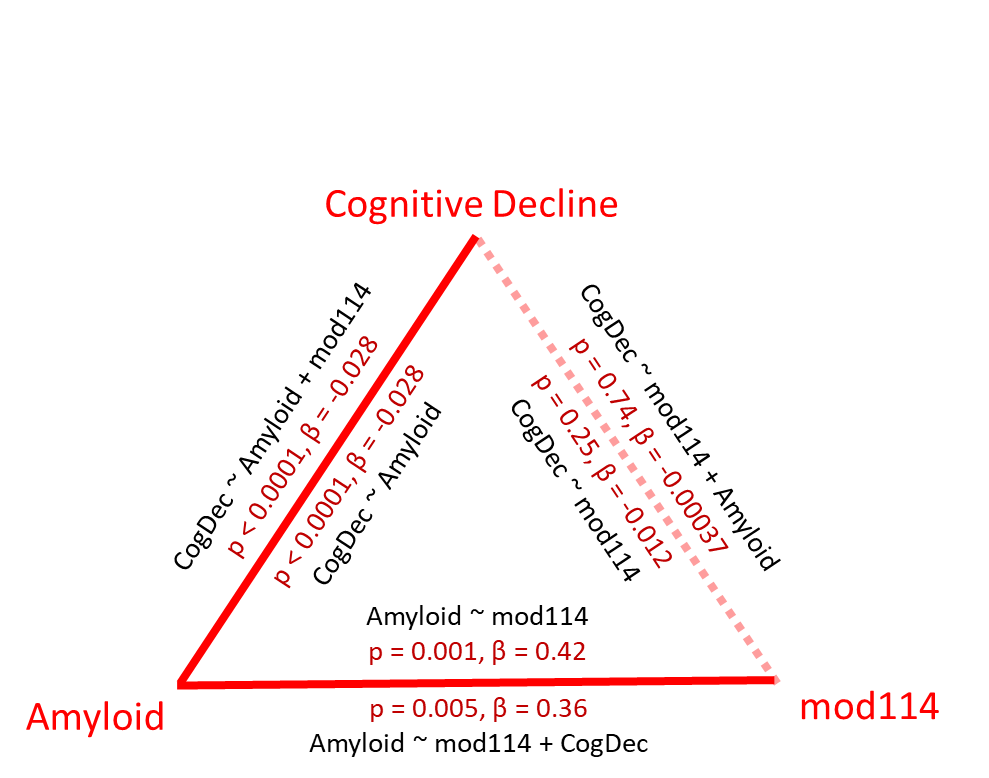

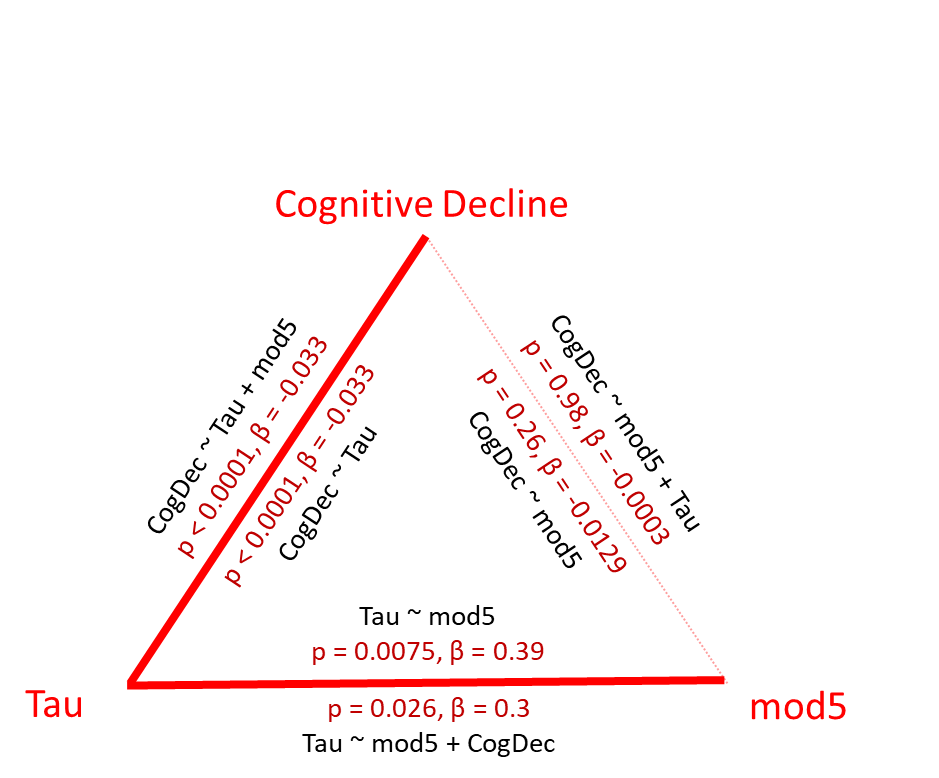

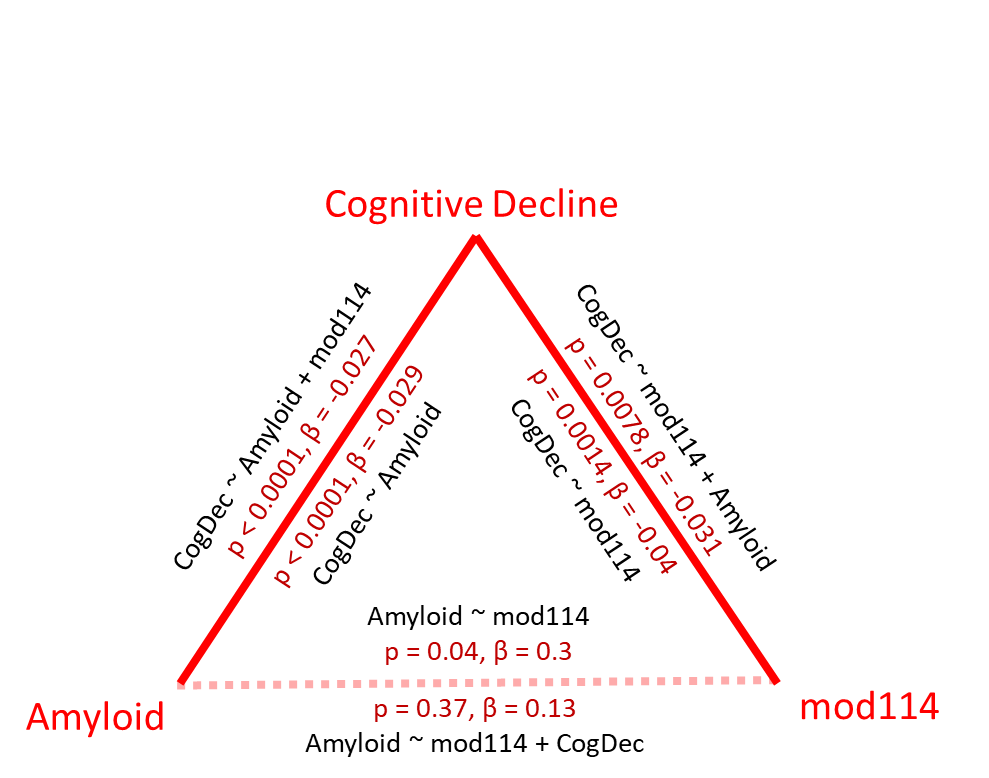

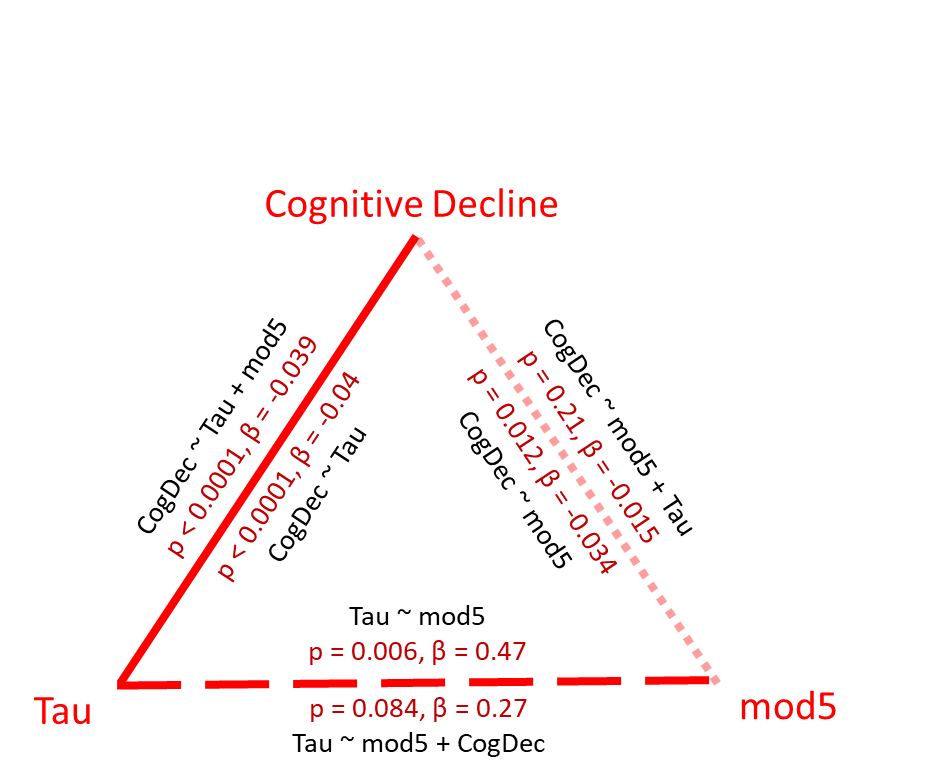

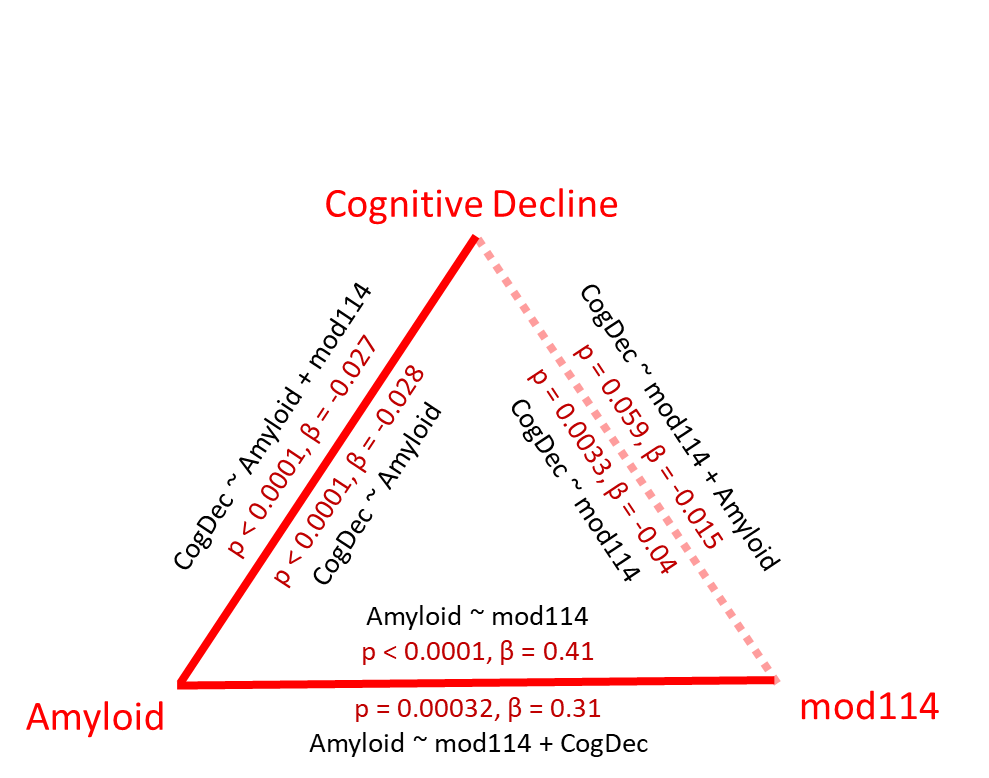


f) All Samples

d) ROS samples

g) ROS samples

e) MAP samples

h) MAP samples

c) All samples

b) All samples

## **Supplementary Figure 8 – Module expression changes for five separate mouse strains.**

Log fold changes for five strains of mouse; HET_TASTPM (heterozygous TASTPM), HO_TASTPM (homozygous TASTPM), TAS10 (APP: K670N/M671L), Tau (MAPT P301L), TPM (PSEN1: M146V) over a time course.

**
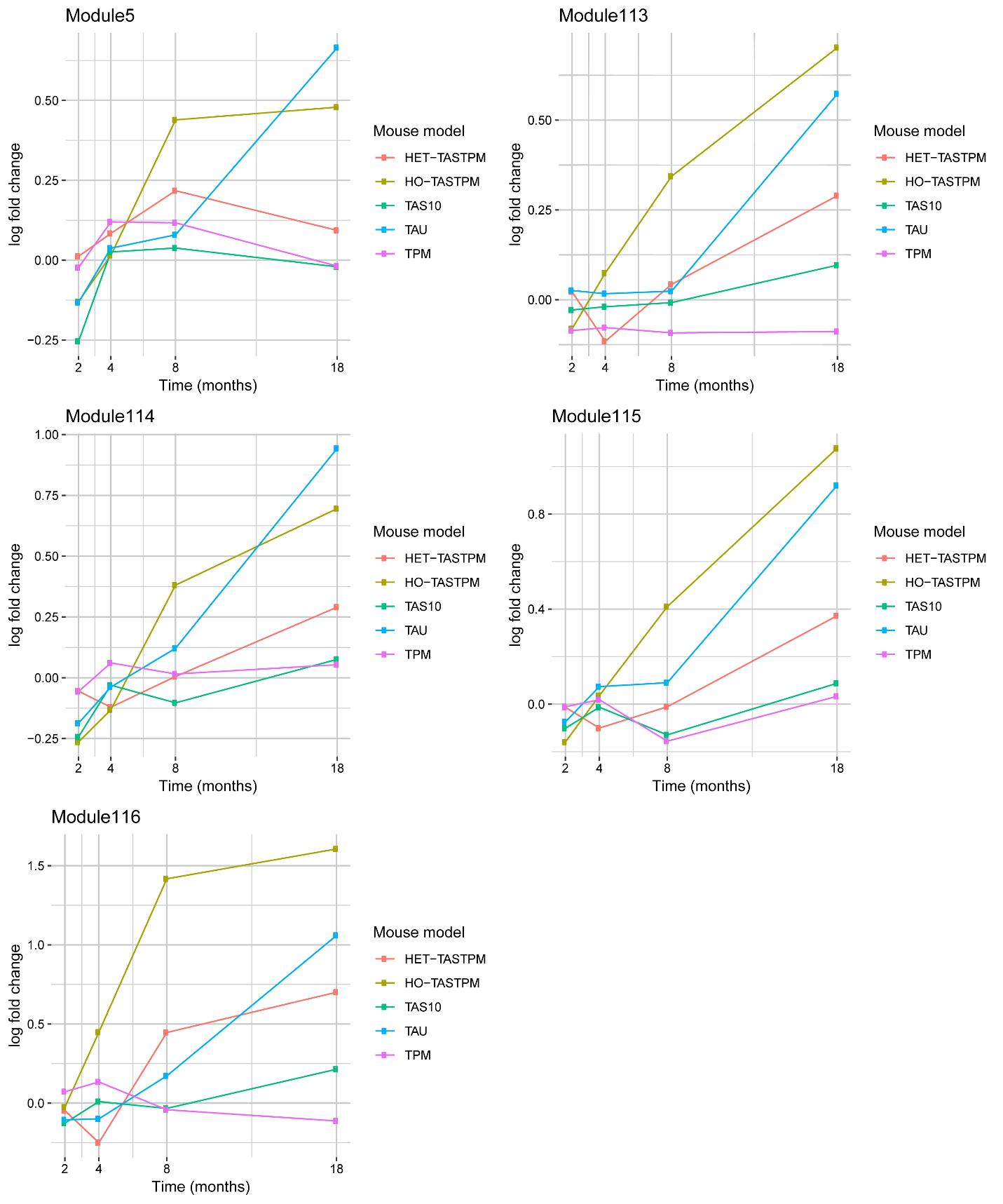
**

## **Supplementary Figure 9 – Module expression in a transgenic mouse model.**

Average standardised module expression of immune cell-type signature gene sets isolated from a transgenic Alzheimer’s disease model^5^. The five human cortical immune modules and their 10 immune cell clusters were investigated. All human cortical immune modules highly expressed the mouse homeostatic microglia (Micoglia1) cluster associated signature genes.


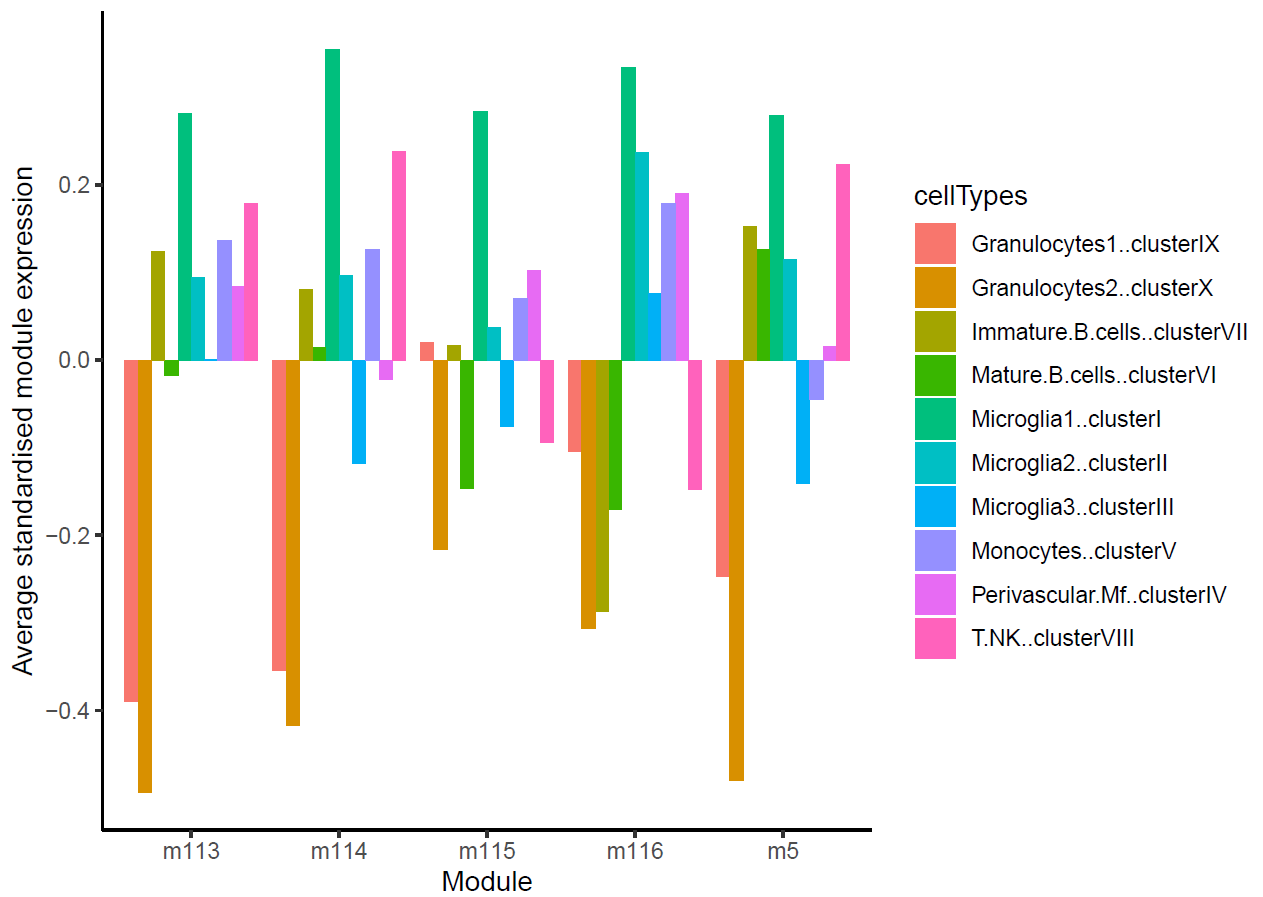


## **Supplementary Figure 10 - Immunostaining showing microglia with stage I, II or III microglia.**

**
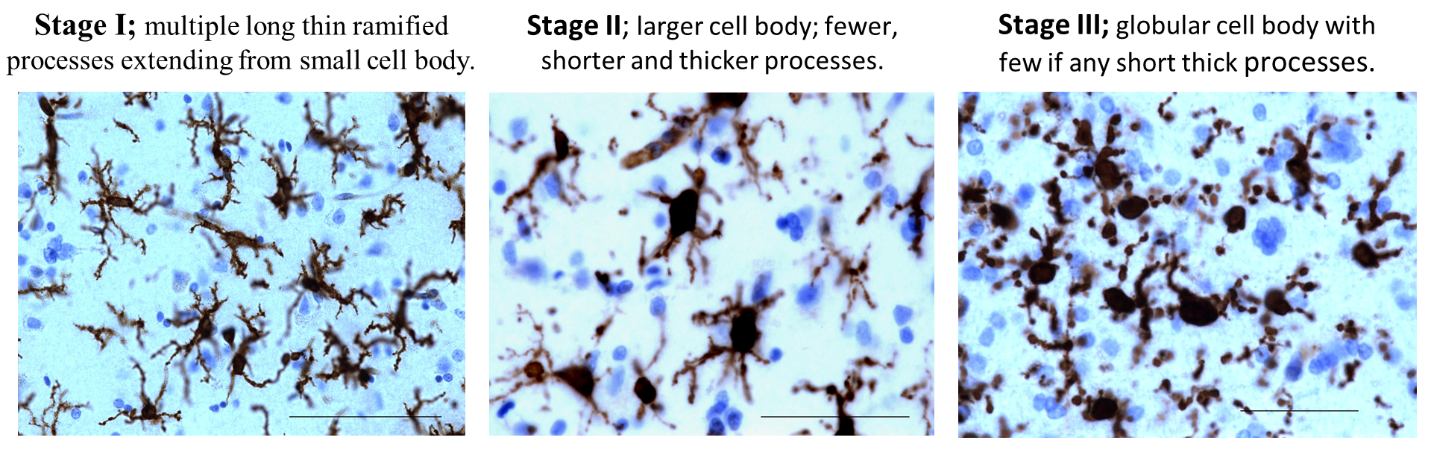
**

## **Supplementary Figure 11 – Module associations in H3K9 acetylation data with activated microglia and cognitive decline.**

Here, we used the module definitions derived from RNA sequence data and impose them on the H3K9Ac data. The t-statistics which capture the associations between H3K9Ac module-level measures with the proportion of Stage III microglia and a decline in cognition are compared. Modules are both colored (red to blue) and sized (big to small) by their enrichment for the microglia signature genes.


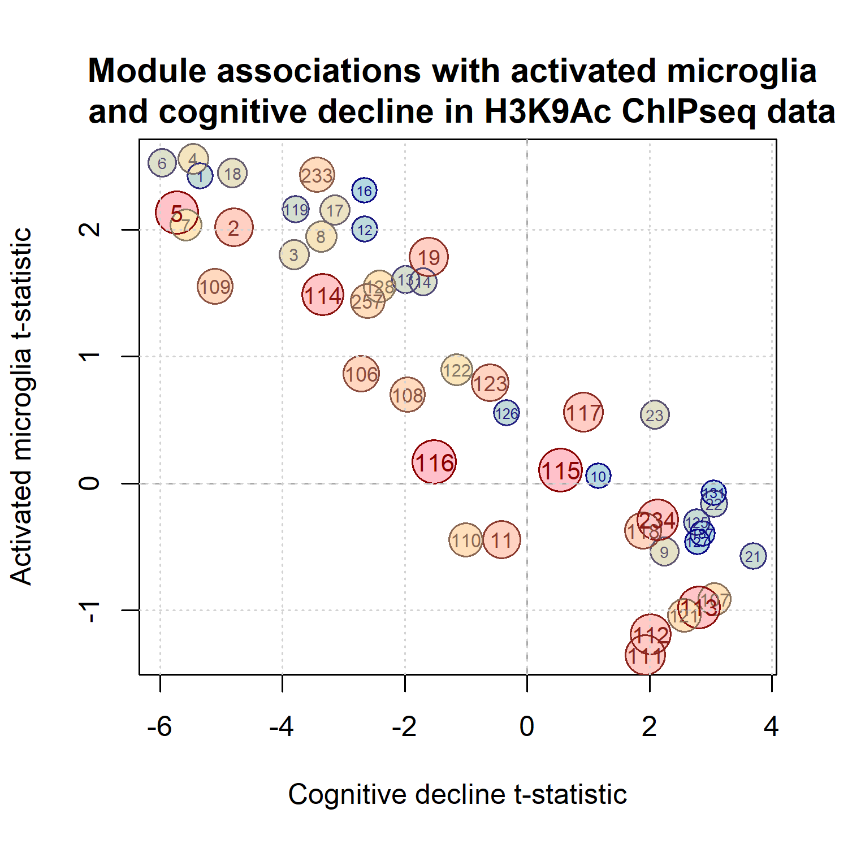


## **Supplementary Figure 12 – Single nucleus sequencing data replicates the association of immune module m5 with Tau pathology.**


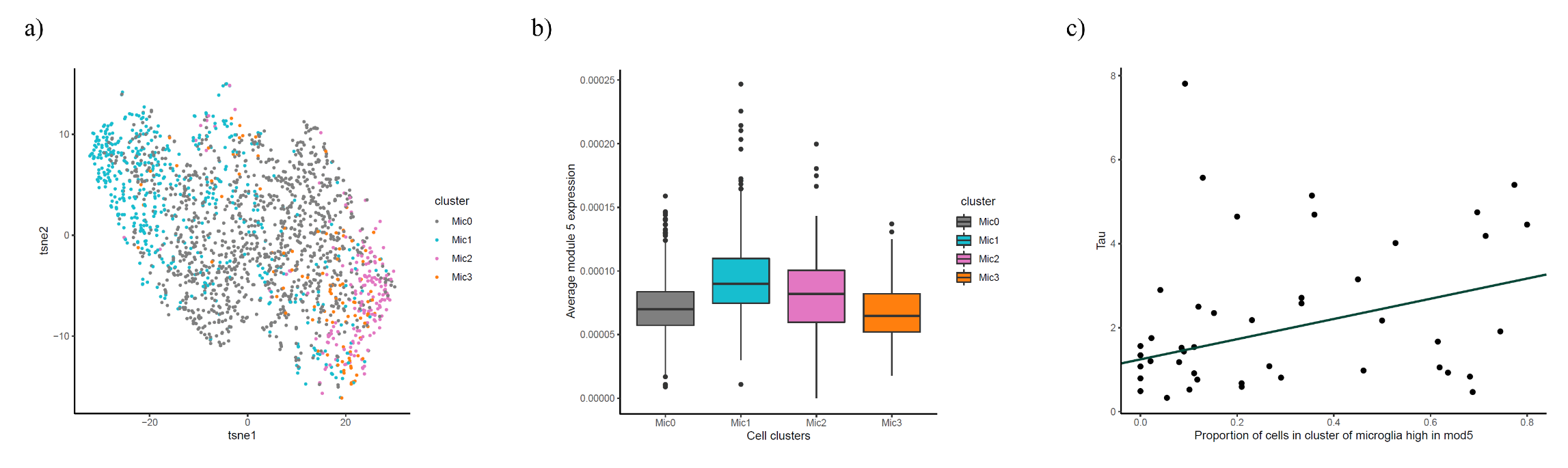
(**a)** A t-SNE plot of microglial cells selected from a snRNA-seq dataset derived from DLPFC. The authors of the original study defined 4 subsets of microglial nuclei (Mic0-3); each dot is one microglia, colored by its cluster assignment. The color key is presented to the right of the panel. Highlighted a four microglia sub-clusters which were identified in the original analysis. **(b)** A boxplot showing the average module 5 expression for the cells in each microglia sub-cluster. **(c)** A scatter plot displaying on the Y axis the level of tau pathology for each subject and on the X axis the proportion of cells in sub-cluster Mic1 relative to the other microglia clusters. The points are coloured by the level of neuritic plaques in each subject (NP). Robust linear regression was used to illustrate the positive relationship (p=0.04, β=-2.2) between the proportion of Mic1 cells and tau pathology. Abbreviations: tsne t-Distributed Stochastic Neighbor Embedding; Mic microglia; NP neuritic plaque.


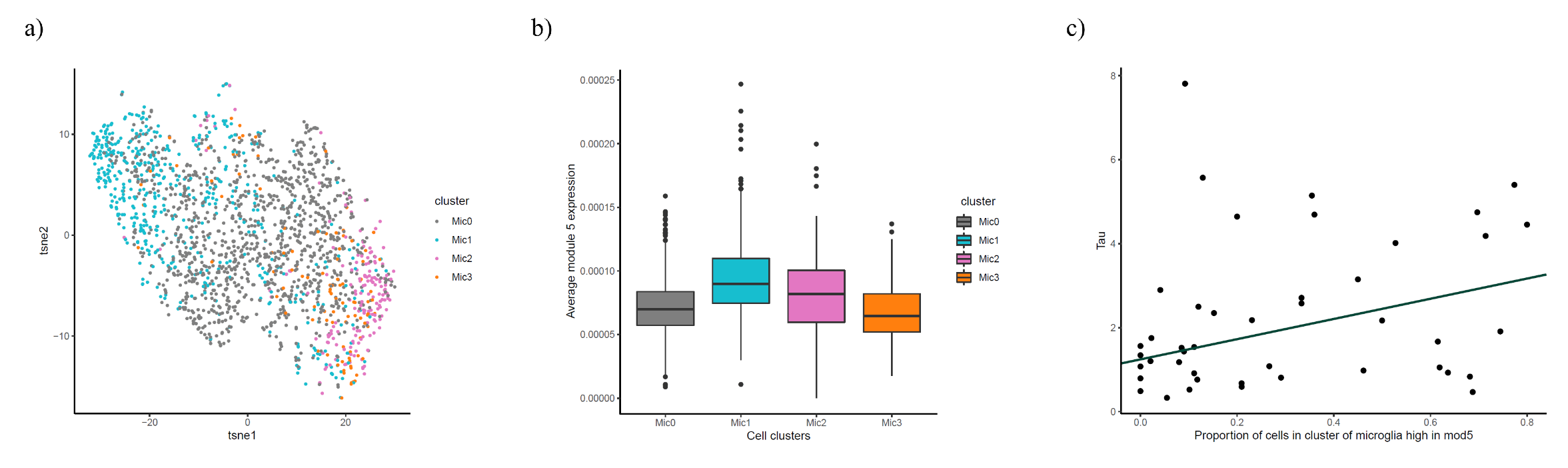


## **Supplementary Figure 13 – Immunofluorescence reveals higher VASP expression relative to TMEM119 with advancing Braak score.**

The average *VASP* expression from immunofluorescence relative to average *TMEM119* expression of cells in each section for six subjects obtained from the Rush Alzheimer’s Disease Center. For each subject Braak, CERAD and NIA-Reagan scores are reported. Subjects are presented in order of increasing Braak stage.


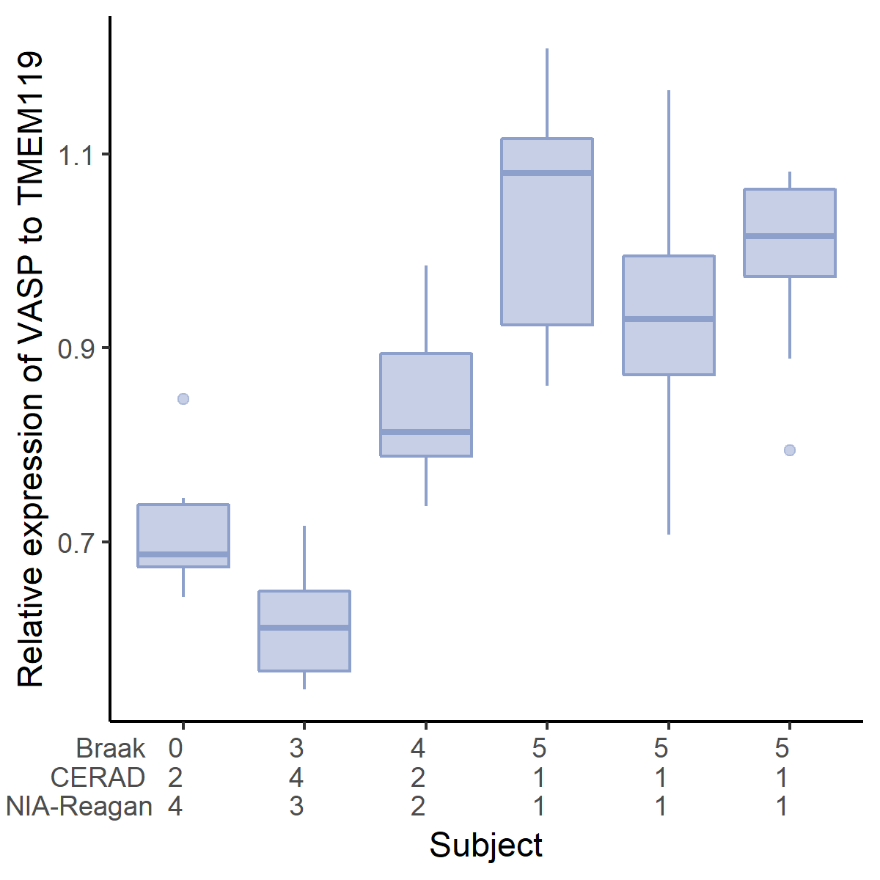


## **Supplementary Figure 14 – Immunofluorescence reveals ACADVL+CD45+ and TRABD+CD45+ cells have a more rounded, activated morphology.**

Both ACADVL and TRABD are proteins encoded by module m5 genes; they are used here as proxies for m5 expression in tissue sections obtained from NYBB to confirm our VASP results (Figure 4) **(a)** Comparison of cellular morphology (compactness - the level of spread or ramification) between ACADVL+ and ACADVL- negative microglia cells (marked by CD45) demonstrates that ACADVL positive microglia are less ramified, when compared to ACADVL- microglia (p=4×10^-14^, β=-0.24, mixed-effects model). **b)** The proportion of ACADVL+ microglia to ACADVL- microglia increases relative to the level of pTau accumulation measured by immunofluorescence in a sister section to the section using in staining ACADVL (p=0.0093, β=0.13, mixed-effects model). **(c)** Comparison of cellular morphology (the level spread or ramification) between TRABD+ and TRABD- negative microglia cells (marked by CD45) demonstrates that TRABD positive microglia are less ramified, when compared to TRABD- microglia (p=3×10^-11^, β=-0.17, mixed-effects model). **d)** The proportion of TRABD+ microglia to TRABD- microglia increases relative to the level of pTau accumulation measured by immunofluorescence in a sister section to the section using in staining TRABD (p=0.011, β=0.11, mixed-effects model). Abbreviations: DLPFC - dorsolateral prefrontal cortex; VASP - vasodilator-stimulated phosphoprotein; CD45 - cluster of differentiation 45, a pan-microglia marker; ACADVL - Acyl-CoA Dehydrogenase Very Long Chain; TRABD - TraB Domain Containing; pTau - phosphorylated tau protein.

**Bibliography**


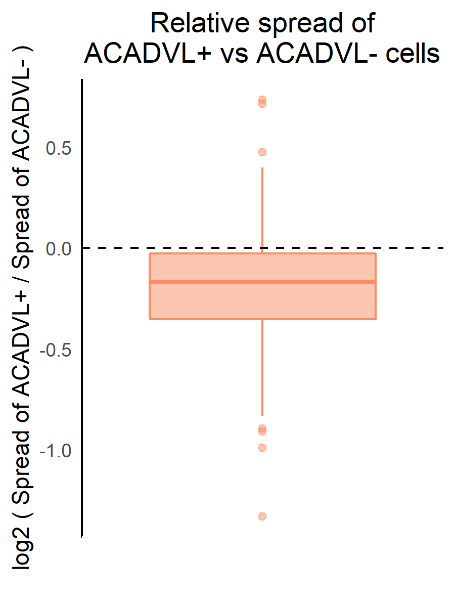

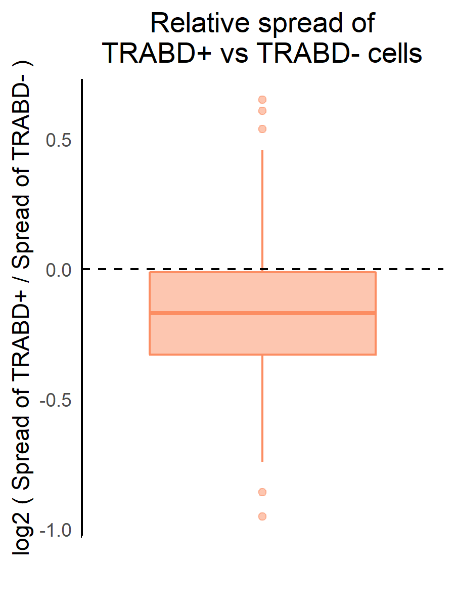

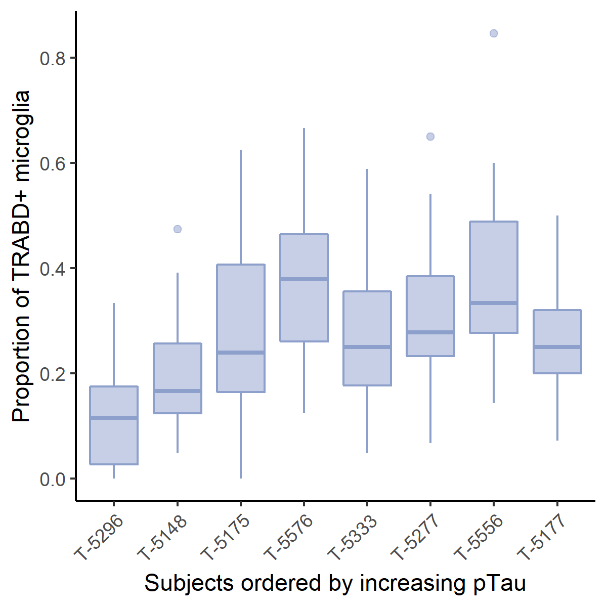

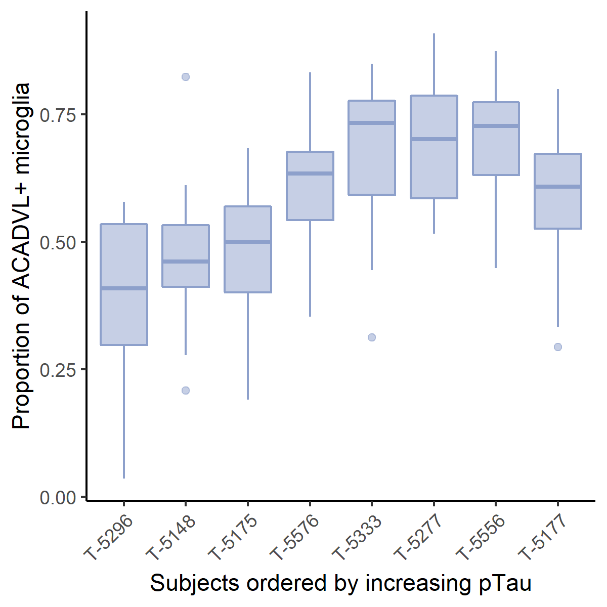


a)

c)

b)

d)

1. Olah, M.*, et al.* A transcriptomic atlas of aged human microglia. *Nature communications* **9**, 539 (2018).

2. Zhang, Y.*, et al.* Purification and characterization of progenitor and mature human astrocytes reveals transcriptional and functional differences with mouse. *Neuron* **89**, 37-53 (2016).

3. Darmanis, S.*, et al.* A survey of human brain transcriptome diversity at the single cell level. *Proceedings of the National Academy of Sciences* **112**, 7285-7290 (2015).

4. Zhang, Y.*, et al.* An RNA-sequencing transcriptome and splicing database of glia, neurons, and vascular cells of the cerebral cortex. *The Journal of neuroscience : the official journal of the Society for Neuroscience* **34**, 11929-11947 (2014).

5. Keren-Shaul, H.*, et al.* A Unique Microglia Type Associated with Restricting Development of Alzheimer's Disease. *Cell* **169**, 1276-1290 e1217 (2017).
